# Brain tyrosinase overexpression implicates age-dependent neuromelanin production in Parkinson’s disease pathogenesis

**DOI:** 10.1038/s41467-019-08858-y

**Published:** 2019-03-07

**Authors:** Iria Carballo-Carbajal, Ariadna Laguna, Jordi Romero-Giménez, Thais Cuadros, Jordi Bové, Marta Martinez-Vicente, Annabelle Parent, Marta Gonzalez-Sepulveda, Núria Peñuelas, Albert Torra, Beatriz Rodríguez-Galván, Andrea Ballabio, Takafumi Hasegawa, Analía Bortolozzi, Ellen Gelpi, Miquel Vila

**Affiliations:** 1 Neurodegenerative Diseases Research Group, Vall d’Hebron Research Institute (VHIR)-Network Center for Biomedical Research in Neurodegenerative Diseases (CIBERNED), 08035 Barcelona, Spain; 2 Telethon Institute of Genetics and Medicine (TIGEM), 80078 Naples, Italy; 3 Department of Neurology, Tohoku University School of Medicine, Miyagi, 980-8574 Japan; 4 Department of Neurochemistry and Neuropharmacology, IIBB–CSIC, August Pi i Sunyer Biomedical Research Institute (IDIBAPS)-Center for Networked Biomedical Research on Mental Health (CIBERSAM), 08036 Barcelona, Spain; 5 Neurological Tissue Bank, Biobanc Hospital Clínic-IDIBAPS, 08036 Barcelona, Spain; 6 Institute of Neurology, Medical University of Vienna, 1090 Vienna, Austria; 7 Department of Biochemistry and Molecular Biology, Autonomous University of Barcelona, 08193 Barcelona, Spain; 8 Catalan Institution for Research and Advanced Studies (ICREA), 08010 Barcelona, Spain

## Abstract

In Parkinson’s disease (PD) there is a selective degeneration of neuromelanin-containing neurons, especially substantia nigra dopaminergic neurons. In humans, neuromelanin accumulates with age, the latter being the main risk factor for PD. The contribution of neuromelanin to PD pathogenesis remains unknown because, unlike humans, common laboratory animals lack neuromelanin. Synthesis of peripheral melanins is mediated by tyrosinase, an enzyme also present at low levels in the brain. Here we report that overexpression of human tyrosinase in rat substantia nigra results in age-dependent production of human-like neuromelanin within nigral dopaminergic neurons, up to levels reached in elderly humans. In these animals, intracellular neuromelanin accumulation above a specific threshold is associated to an age-dependent PD phenotype, including hypokinesia, Lewy body-like formation and nigrostriatal neurodegeneration. Enhancing lysosomal proteostasis reduces intracellular neuromelanin and prevents neurodegeneration in tyrosinase-overexpressing animals. Our results suggest that intracellular neuromelanin levels may set the threshold for the initiation of PD.

## Abstract (web summary)

It is unclear if neuromelanin plays a role in Parkinson’s disease pathogenesis since common laboratory animals lack this pigment. Authors show here that overexpression of human tyrosinase in the substantia nigra of rats resulted in an age-dependent production of human-like neuromelanin within nigral dopaminergic neurons and is associated with a Parkinson’s disease phenotype when allowed to accumulate above a specific threshold.

## Introduction

In Parkinson’s disease (PD), neurons that contain the dark-brown cytoplasmic pigment neuromelanin (NM) are particularly susceptible to neurodegeneration^1^. Indeed, while PD patients exhibit an abnormal accumulation of alpha-synuclein (aSyn) protein in different brain regions, and even in peripheral tissue^2^, neurodegeneration preferentially occurs within NM-containing regions in these patients^3–5^. In contrast, neuronal loss in non-melanized brain regions is either inconsistent, not specific to PD, or secondary to the loss of interconnected NM-containing neurons^3–5^. Such highly vulnerable NM-containing brain regions include the substantia nigra pars compacta (SNpc), where the loss of dopaminergic (DA) neurons therein leads to the typical motor symptoms of the disease and constitutes the cardinal pathologic diagnostic criterion for PD. In the human SNpc, which is the primary source of NM in the human brain, NM levels are actually so high that this structure can be seen macroscopically as a darkened area (hence the origin of the name given to this brain region)^6^. NM is restricted to catecholamine-producing regions and forms only in neurons. It first becomes observable in the human SNpc at ~3 years of age and progressively accumulates over time within the cells in which it has been produced, as neurons apparently lack the mechanisms for degrading or eliminating this pigment. As a consequence, intracellular NM builds up with age until occupying most of the neuronal cytoplasm^7^. Importantly, aging is the main risk factor for developing PD^8^.

DA-producing cell groups of the normal human midbrain differ markedly from each other in terms of the percentage of NM-pigmented neurons they contain^1,9,10^. In PD, the estimated cell loss in these cell groups directly correlates with the percentage of NM-pigmented neurons normally present in them^1,9,10^. Likewise, within each cell group in PD brains, there is greater relative sparing of weakly pigmented than of strongly melanized neurons^1,9,10^. Also, classical Lewy bodies (LB), i.e. aSyn-containing intracytoplasmic inclusion bodies that represent the pathological hallmark of the disease, as well as their presumed precursor structures, pale bodies (PB), typically appear within the intracellular areas of the cytoplasm in which NM accumulates and form in close physical association with this pigment^11^. Along this line, studies in human brains have shown that aSyn redistributes to the lipid component of NM at early PD stages^12^ and that aSyn becomes entrapped within NM granules extracted from PD, but not control, brains^13^. Further linking PD neuropathology with NM, PD-linked neuroinflammatory changes are highly localized within NM-containing areas and are barely observed in non-melanized regions, such as the cortex, despite the latter exhibiting PD-related aSyn depositions^14^.

According to the above observations, PD pathogenesis appears inextricably linked to the presence of NM. However, despite the close and long-established association between NM and PD, the physiological significance of NM and its potential contribution to PD pathogenesis remain unknown. The current lack of knowledge about the role of NM both in healthy subjects and in PD patients lies in the fact that, in contrast to humans, laboratory animal species commonly used in experimental research, such as rodents, lack NM^15^. In fact, the great abundance of NM in the brainstem is unique to humans, as macroscopic dark pigmentation of this brain area is not observed in other animal species^16^. Consequently, a factor so intimately linked to PD such as NM has been surprisingly neglected so far in experimental in vivo paradigms of the disease. To address this major limitation, in the present study we generate the first rodent model of age-dependent human-like NM production in SNpc DA neurons at levels up to those reached in elderly humans. Using this unique model, we show that progressive intracellular NM accumulation above a specific threshold is associated with a PD phenotype.

## Results

### Age-dependent NM formation in tyrosinase-overexpressing rats

The mechanism of synthesis of NM is poorly understood, but it is generally accepted that NM is formed as an inert cellular by-product of DA synthesis produced by simple non-enzymatic DA autoxidation^17^. In contrast, it is well-established that the synthesis of peripheral melanins (e.g. skin and hair), which occurs within specialized cells (i.e. melanocytes), results from an enzymatically driven biosynthetic pathway initiated with the hydroxylation of l-tyrosine to l-DOPA, followed by the oxidation of l-DOPA to the melanin precursor DOPAquinone, in which tyrosinase is the key, rate-limiting enzyme^18,19^. Remarkably, tyrosinase expression and activity are not apparently restricted to melanocytes but have also been observed, although at low levels, in the brain, including human SNpc^20–22^ (Supplementary Figure 1). In addition, tyrosinase is also able to oxidize the catechol ring of DA, which is an essential event required for NM synthesis^19^. However, whether brain tyrosinase may actually contribute to NM synthesis is currently unknown. Here we assessed what are the consequences of overexpressing tyrosinase in the SNpc of rodents.

**Fig. 1:**
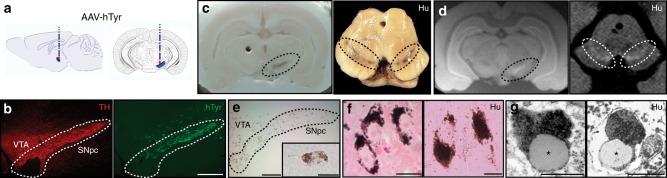
Human-like NM production in AAV-hTyr-injected rats. **a** Schematic representation of the site of AAV-hTyr unilateral stereotaxic injection above the SNpc of the rat brain. **b** Representative photomicrographs of a 30-μm-thick ipsilateral SNpc section from an AAV-hTyr-injected rat (1 m post-AAV injection) immunostained for TH (red) and hTyr (green). Scale bar, 500 μm. **c** Left, representative unstained AAV-hTyr-injected rat brain (2 m post-AAV injection) mounted in a cryostat in which ipsilateral SNpc can be detected macroscopically as a brown, darkened area (dashed outline). A hole was made in the contralateral hemisphere as anatomical reference. Right, representative unstained midbrain from a 62-year-old human control subject (Hu) in which the SNpc can be detected macroscopically (bilateral dashed outlines). **d** Representative NM-sensitive high-resolution T1-weighted magnetic resonance imaging of an AAV-hTyr-injected rat brain at 2 m post-AAV injection (left, ex-vivo) and of a 59-year-old human control brain (Hu, right, in vivo). SNpc can be detected as a unilateral (AAV-hTyr-injected rodent) or bilateral (human) hyperintense area (dashed outlines). **e** Representative photomicrograph of an unstained 30-μm-thick ipsilateral SNpc section from an AAV-hTyr-injected rat (2 m post-AAV injection) in which NM is shown in brown. Inset, high magnification of a melanized neuron. Scale bars, 100 μm and 12.5 μm (inset). **f** Representative photomicrographs of Masson-Fontana melanin staining (NM in dark brown) in 5-μm-thick SNpc sections from an AAV-hTyr-injected rat at 2 m post-AAV injection (left) and an 80-year-old human control subject (Hu, right). Scale bars, 25 μm (left) and 12.5 μm (right). **g** Representative electron micrograph of NM granules in the ipsilateral SNpc of an AAV-hTyr-injected rat at 4 m post-AAV injection (left) and a 75-year-old human control subject (Hu, right). NM pigment is detected as an electron dense matrix. Characteristic associated lipid droplets are indicated with asterisks. Scale bars, 500 nm

Adult rats received a single unilateral stereotaxic injection of an adeno-associated viral (AAV) vector expressing human tyrosinase (hTyr) above the right SNpc (Fig. 1a). By 2–4 weeks (w) post-AAV-hTyr injection, up to ~80–90% of ipsilateral SNpc DA neurons were transduced and expressed the hTyr protein (Fig. 1b). By 2 months (m) post-AAV-hTyr injection, the ipsilateral SNpc from these animals could be visualized macroscopically, in the absence of any staining, as a darkened brown area, similar to human melanized SNpc tissue (Fig. 1c). In contrast, the contralateral SNpc from the same animals could not be detected macroscopically, as rodents lack NM (Fig. 1c). Similar to humans, ipsilateral SNpc from AAV-hTyr-injected animals could also be detected macroscopically as a hyperintense area by NM-sensitive high-resolution T1-weighted magnetic resonance imaging (Fig. 1d). Optical microscopy visualization confirmed that the darkened SNpc area in AAV-hTyr-injected rats corresponded to the presence of an intracellular dark-brown fine granular pigment analogous to human NM within ipsilateral SNpc neurons (Fig. 1e). Virtually all (~97%) hTyr-expressing SNpc DA neurons from AAV-hTyr-injected animals produced NM (Supplementary Figure 2). As in humans, NM from AAV-hTyr-injected rats stained prominently with the melanin marker Masson-Fontana, which reflects the ability of NM to chelate metals (Fig. 1f). Ultrastructural examination by electron microscopy identified NM granules from AAV-hTyr-injected rats as membrane-delimited autophagic structures with an electron-dense matrix of irregular shape and size, associated with characteristic lipid droplets, equivalent to what has been described in humans^23^ (Fig. 1g). Within the SNpc, NM pigment in AAV-hTyr-injected rats was restricted to TH^−^immunopositive DA neurons (Supplementary Figure 2), thereby mimicking the pattern of human NM distribution within catecholamine-producing neurons^6^.

**Fig. 2:**
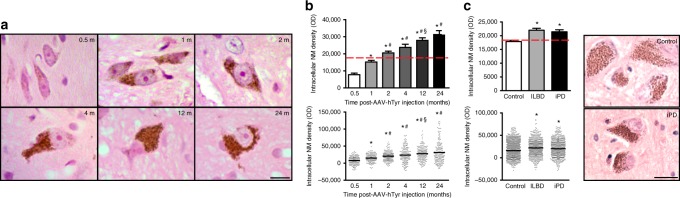
Age-dependent NM accumulation in AAV-hTyr-injected rats. **a** Hematoxylin-eosin (H&E)-stained brain sections showing progressive intracellular NM accumulation (brown) within ipsilateral SNpc DA neurons from AAV-hTyr-injected rats. Scale bar, 12.5 μm. **b** Quantification of intracellular NM optical density in ipsilateral SNpc DA neurons of AAV-hTyr-injected rats. *p < 0.05, compared to 0.5 m; #*p* < 0.05, compared to 1 m; §*p* < 0.05, compared to 2&4 m. **c** Left, quantification of intracellular NM optical density in post-mortem SNpc sections from elderly human (average 80 years old) control subjects, age-matched ILBD subjects and age-matched idiopathic PD patients. *p < 0.05, compared to control subjects. Right, H&E-stained human brain sections. Scale bar, 12.5 μm. In **b**, **c** values are mean ± SEM. In **b**, *n* = 163 neurons from *n* = 7 rats (0.5 m), *n* = 136 neurons from *n* = 5 rats (1 m), *n* = 174 neurons from *n* = 5 rats (2 m), *n* = 162 neurons from *n* = 7 rats (4 m), *n* = 172 neurons from *n* = 6 rats (12 m), *n* = 179 neurons from *n* = 6 rats (24 m). In **c**, *n* = 1436 neurons from *n* = 6 control subjects, *n* = 640 neurons from *n* = 3 ILBD subjects, *n* = 644 neurons from *n* = 10 PD subjects. See Supplementary Table 1 for additional information on the human subjects used for the analyses in **c**. Statistical analyses: ANOVA on ranks; Dunn’s post-hoc test. Photomicrographs correspond to 5-μm-thick sections

In human SNpc DA neurons, NM is continuously produced throughout life and progressively accumulates with age until it occupies a major portion of the neuronal cytoplasm^24^. Similarly, quantification of intracellular NM optical density in SNpc neurons of AAV-hTyr-injected rats, which reflects the actual concentration of NM^7,10^, revealed that intracellular NM levels steadily increase in the SNpc of these animals over time, from 0.5 up to 24 m post-AAV-hTyr injection, until occupying most of the neuronal cytoplasm (Fig. 2a, b). In ~1–2 m, intracellular NM in AAV-hTyr-injected rats reached levels equivalent to those in post-mortem SNpc tissue from elderly (~80 years old) human control subjects (Fig. 2b, c). In ~2–4 m, AAV-hTyr-injected rats exhibited intracellular NM levels equivalent to those in post-mortem SNpc tissue from (i) subjects with incidental LB disease (ILBD), i.e. clinically healthy individuals exhibiting LB pathology at autopsy who are considered to represent early, presymptomatic stages of PD, and (ii) established PD patients (Fig. 2b, c), in both of which intracellular NM levels appeared significantly higher than those from age-matched control subjects (Fig. 2c; see also Supplementary Table 1).

Overall, these results show that hTyr expression in rat SNpc leads to the production of human-like NM within SNpc DA neurons. As in humans, intracellular NM levels in these animals steadily increase over time, reaching first levels equivalent to those of elderly human brains and further increasing up to levels attained in pre-PD subjects and PD patients.

### Progressive PD-like neurodegeneration in NM-producing rats

**Fig. 3:**
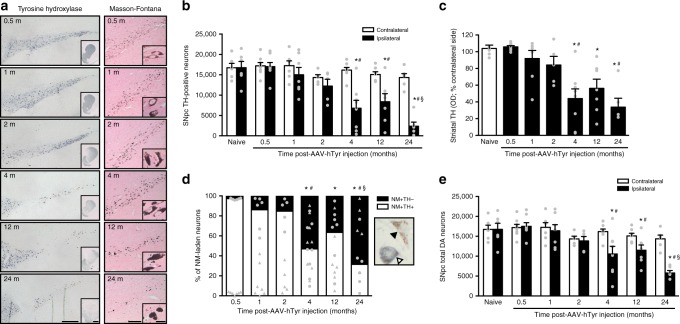
Progressive nigrostriatal degeneration in AAV-hTyr-injected rats. **a** Left, ipsilateral SNpc and striatum (inset) sections immunostained for TH (blue). Right, ipsilateral SNpc sections stained with Masson-Fontana (NM in dark brown; inset, higher magnification of melanized neurons). Scale bars, 300 μm (left), 2 mm (left inset), 150 μm (right), and 12.5 μm (right inset). **b** Stereological cell counts of SNpc TH-positive neurons in AAV-hTyr-injected rats. *p < 0.05, compared to respective contralateral (non-injected) side; #*p* ≤ 0.05, compared to ipsilateral naive, 0.5, 1, and 2 m; §*p* < 0.05, compared to ipsilateral 4 and 12 m (two-way ANOVA; Student–Newman–Keuls post-hoc test). **c** Optical densitometry of striatal TH-positive fibers in AAV-hTyr-injected rats. *p < 0.05, compared to 0.5 m; #*p* < 0.05, compared to naive (ANOVA on ranks; Dunn’s post-hoc test). **d** Stereological cell counts of SNpc NM + /TH + (white arrowhead) and NM + /TH− (black arrowhead) neurons vs total NM + neurons in AAV-hTyr-injected rats. *p < 0.05, compared to 0.5 m; #*p* < 0.05, compared to 1 and 2 m; §*p* < 0.05, compared to 12 m (one-way ANOVA; Student–Newman–Keuls post-hoc test). **e** Stereological cell counts of total SNpc DA neurons (including TH-immunopositive and TH-immunonegative melanized neurons) in AAV-hTyr-injected rats. *p < 0.05, compared to respective contralateral side; #*p* < 0.05, compared to ipsilateral naive, 0.5 and 1 m; §*p* < 0.05, compared to ipsilateral 2, 4, and 12 m (two-way ANOVA; Student–Newman–Keuls post-hoc test). In all panels, values are mean ± SEM. In **a**–**e**, *n* = 6 (Naïve), *n* = 8 (0.5 m), *n* = 7 (1 m), *n* = 5 (2 m), *n* = 8 (4 m), *n* = 7 (12 m), and *n* = 6 (24 m). Photomicrographs correspond to 5-μm-thick sections

Using our newly generated NM-producing rats, we next assessed whether age-dependent intracellular NM accumulation, up to levels reached in elderly subjects and ILBD/PD cases, may be associated to compromised cell viability. Stereological cell counts of SNpc TH-positive neurons in AAV-hTyr-injected rats revealed a progressive, age-dependent loss of TH-positive SNpc neurons, starting by 4 m post-AAV-hTyr injection (Fig. 3a, b). Progressive loss of SNpc TH-positive neurons in AAV-hTyr-injected rats was accompanied by a reduction of striatal DA TH-positive fibers, as measured by optical densitometry (Fig. 3c). Loss of nigrostriatal DA fibers in AAV-hTyr-injected rats was accompanied by decreased striatal DA levels, as measured by high-performance liquid chromatography (HPLC) (Supplementary Table 2), and abnormally enlarged nigrostriatal TH-positive nerve terminals (Supplementary Fig. 3).

Similar to PD brains, NM-laden neurons from AAV-hTyr-injected rats also exhibited an early phenotypic loss of TH expression, as indicated by an increased percentage of TH-immunonegative neurons within the total population of NM-containing neurons (Fig. 3d). TH-immunonegative pigmented neurons are believed to represent dysfunctional/dying SNpc DA neurons^1^. To distinguish between reduced TH expression and actual cell death, we performed stereological cell counts of total SNpc DA neurons (including TH-immunopositive and TH-immunonegative melanized SNpc neurons), which confirmed a progressive loss of SNpc DA neurons in AAV-hTyr-injected rats (Fig. 3e). In parallel to the loss of melanized SNpc neurons, there was a progressive depigmentation of the SNpc in AAV-hTyr-injected rats, as it occurs in PD patients (Fig. 3a, right panel). Correlation analyses in AAV-hTyr-injected rats confirmed a strong positive and significant correlation between intracellular NM levels and SNpc DA neurodegenerative changes (Supplementary Figure 3). In contrast, no degeneration of non-DA, non-melanized neighboring gamma-aminobutyric acid nigral neurons was observed in AAV-hTyr-injected rats, as indicated by the preservation of the ipsilateral gamma-aminobutyric acidergic nigrotectal pathway in these animals (Supplementary Figure 4), despite this region being systematically transduced with hTyr in AAV-hTyr-injected animals, due to its proximity to the site of the AAV-hTyr injection (Supplementary Figure 2). Relevant to humans, these neurons do not degenerate in PD patients^3^ and do not contain NM in humans, as they do not use a catecholaminergic NM precursor as neurotransmitter. In addition, no nigrostriatal DA degeneration was observed in aging non-melanized contralateral SNpc from AAV-hTyr-injected rats (Fig. 3b, e) nor in aging non-melanized ipsilateral SNpc from AAV-empty vector (EV)-injected or vehicle-injected control rats (Supplementary Figure 4).

**Fig. 4:**
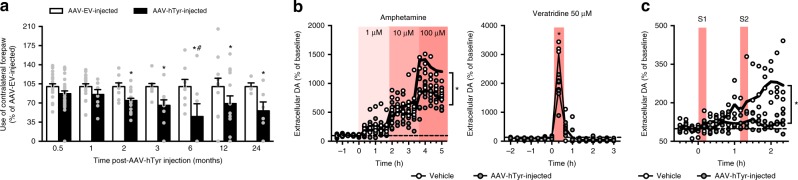
Early dopaminergic dysfunction in AAV-hTyr-injected rats. **a** Contralateral forepaw use in AAV-hTyr- and AAV-EV-injected rats, as assessed with the cylinder test. *p ≤ 0.05, compared to AAV-EV-injected animals at the same time-point; #*p* < 0.05, compared to 0.5 m AAV-hTyr animals (two-way ANOVA; Student–Newman–Keuls post-hoc test). **b** Striatal DA release in AAV-hTyr- and vehicle-injected rats measured by microdialysis in the ipsilateral striatum following local amphetamine (left) or veratridine (right) administration by reverse-dialysis. *p < 0.05, compared to vehicle-injected animals (ANOVA for repeated measures of the DA values during the specified time periods; Tukey’s post-hoc test). Baseline DA concentration (fmol/fraction-20 min): amphetamine experiments, 82.83 ± 19.16 (vehicle) vs 94.38 ± 21.82 (AAV-hTyr); veratridine experiments, 25.52 ± 4.33 (vehicle) vs 29.93 ± 6.46 (AAV-hTyr). **c** Striatal DA release in AAV-hTyr- and vehicle-injected rats measured by microdialysis in the ipsilateral striatum following electrical stimulation of the MFB for 10-min periods under S1 and S2 conditions (S1, 2.0 Hz, 0.1 mA, 0.2 ms; S2, 10 Hz, 0.1 mA, 1 ms). *p < 0.05, compared to stimulated AAV-hTyr-injected rats (ANOVA for repeated measures; Tukey’s post-hoc test). Baseline DA concentration (fmol/fraction-10 min): 16.8 ± 2.38 (vehicle) vs 23.02 ± 6.37 (AAV-hTyr). In all panels, values are mean ± SEM. In **a**, AAV-EV-injected rats *n* = 20 (0.5 m), *n* = 20 (1 m), *n* = 9 (2 m), *n* = 9 (3 m), *n* = 8 (6 m), *n* = 12 (12 m), *n* = 4 (24 m); and AAV-hTyr-injected rats *n* = 29 (0.5 m), *n* = 8 (1 m), *n* = 21 (2 m), *n* = 14 (3 m), *n* = 7 (6 m), *n* = 14 (12 m), *n* = 6 (24 m). In **b**, **c**, experiments were performed at 1-2 m post-AAV injection. In **b**, *n* = 6 vehicle-injected, *n* = 6 AAV-hTyr-injected rats + Amphetamine and *n* = 8 AAV-hTyr-injected rats + Veratridine. In **c**, *n* = 4 animals per group

We next determined whether the neurodegenerative changes observed in AAV-hTyr-injected rats were accompanied by functional alterations. At different time-points post-AAV-hTyr injections, from 0.5 up to 24 m, rats were subjected to behavioral analyses with the cylinder test, which allows asymmetrical alterations in nigrostriatal DA function to be detected^25^. Concomitant with the ipsilateral degeneration of NM-laden SNpc neurons reported above, AAV-hTyr-injected rats exhibited contralateral forepaw hypokinesia, as indicated by reduced contralateral forepaw use, compared to AAV-EV-injected animals (Fig. 4a). These motor alterations started by 2 m post-AAV-hTyr injection, thus coinciding with the first signs of striatal axonal damage (i.e. axonal swelling, Supplementary Figure 3) and preceding nigrostriatal neurodegeneration in these animals (Fig. 3a–e). As motor impairment preceded overt neuron cell death in AAV-hTyr-injected rats, we next evaluated whether striatal DA function was already impaired in these animals at such early stages. Striatal DA release following chemical or electrical stimulation was measured by microdialysis in the ipsilateral striatum of vehicle- and AAV-hTyr-injected rats. At baseline, no differences in extracellular striatal DA concentration, measured by HPLC, were observed between control- and AAV-hTyr-injected freely moving rats, indicating that hTyr expression per se does not alter basal DA neurotransmission (Fig. 4b). Stimulation of striatal DA release by local amphetamine administration induced concentration-dependent increases of extracellular striatal DA in both control- and AAV-hTyr-injected rats, but this effect was much less pronounced in the latter group of animals (Fig. 4b). Similarly, when stimulated with the depolarizing agent veratridine, striatal DA release was also less prominent in AAV-hTyr-injected rats compared to control-injected animals (Fig. 4b). We next assessed striatal DA release induced by electrical stimulation of the ipsilateral medial forebrain bundle (MFB) via an electrode implanted in these animals. Similar to freely moving rats, no differences in baseline extracellular DA levels in the striatum were detected between control- and AAV-hTyr-injected anaesthetized animals (Fig. 4c). However, following electrical stimulation of the MFB, AAV-hTyr-injected animals exhibited a markedly reduced release of striatal DA compared to control-injected rats (Fig. 4c). These results indicate that synaptic DA release by NM-laden nigrostriatal neurons is impaired before any overt SNpc cell loss has occurred, as is believed to be the case in PD patients^26,27^.

To further corroborate the occurrence of a synaptic failure in these animals, we also measured the striatal densities of dopamine transporter (DAT) and vesicular monoamine transporter 2 (VMAT2) immunopositive fibers in AAV-hTyr-injected rats at different times post-AAV-hTyr injections, from 0.5 up to 24 m (Supplementary Figure 3). Striatal DAT-positive fibers in AAV-hTyr-injected rats exhibited an earlier and more extensive loss than striatal TH-positive fibers, starting by 2 m post-AAV-hTyr injections (i.e. at the time of neuronal dysfunction but before the occurrence of overt neurodegeneration in these animals) and reaching up to a 90% striatal depletion. In contrast, striatal VMAT2 levels were significantly decreased by 12 m post-AAV-hTyr injections and reached a 50% reduction. These results concur with equivalent neuroimaging in vivo analyses in PD patients, in which neuroimaging reductions in DAT striatal density (i.e. by [^11^C]d-threo-methylphenidate PET or ^123^I-2β-carbomethoxy-3β-(4-iodophenyl)-N-(3-fluoropropyl)-nortropane SPECT) can be detected at early stages of the disease, including in pre-motor idiopathic REM sleep behavior disorder patients^28–30^ and pre-symptomatic LRRK2 non-manifesting carriers^31–34^. In contrast, neuroimaging reductions in VMAT2 striatal density (i.e. by [^11^C]( ± )dihydrotetrabenazine PET) only become consistent once the disease is fully established^35–38^, probably as a compensatory mechanism to maintain extracellular levels of DA at early disease stages and thus delay the onset of parkinsonian symptoms^37^.

**Fig. 5:**
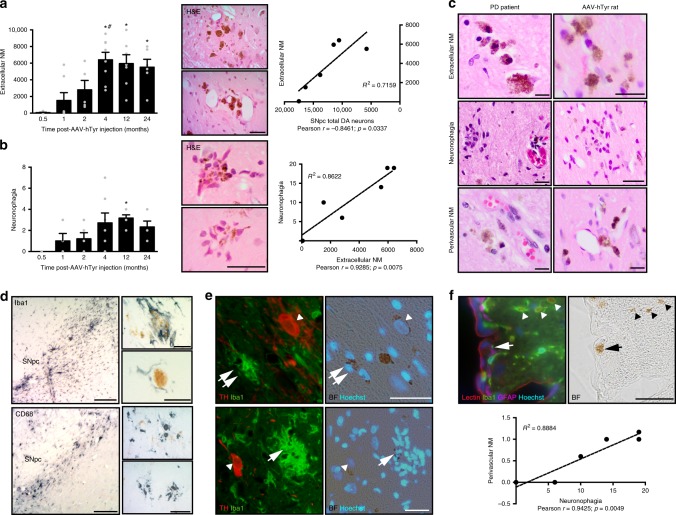
Extracellular NM and neuronophagia in AAV-hTyr-injected rats. **a** Left, extracellular NM aggregates in ipsilateral SNpc from AAV-hTyr-injected rats. *p < 0.05, compared to 0.5&1 m; #*p* < 0.05, compared to 2 m (one-way ANOVA; Student–Newman–Keuls post-hoc test). Middle, hematoxylin-eosin (H&E)-stained ipsilateral SNpc sections from AAV-hTyr-injected rats (4 m). Scale bar, 25 μm. Right, Pearson correlation analysis between total SNpc DA neurons and extracellular NM. **b** Left, neuronophagia in ipsilateral SNpc from AAV-hTyr-injected rats. *p < 0.05, compared to 0.5 m (ANOVA on ranks; Dunn’s post-hoc test). Middle, H&E-stained ipsilateral SNpc sections from AAV-hTyr-injected rats (4 and 12 m). Scale bar, 62.5 μm. Right, Pearson correlation analysis between extracellular NM and neuronophagia. **c** H&E-stained midbrain sections from PD post-mortem brains and AAV-hTyr-injected rats. Scale bars, 12.5 μm (top & bottom) and 25 μm (middle). **d** Ipsilateral SNpc sections from AAV-hTyr-injected rats (2–12 m) immunostained for the microglial marker Iba1 (blue, top) or the macrophage marker CD68 (blue, bottom). Scale bars, 375 μm (Iba1, low magnification), 12.5 μm (Iba1, high magnification), 150 μm (CD68, low magnification), and 25 μm (CD68, high magnification). **e** Ipsilateral SNpc sections from AAV-hTyr-injected rats (4 m) immunostained for TH (red) and Iba1 (green) and co-stained with the nuclear marker Hoechst (blue). Arrowheads, intact TH-positive NM-laden neurons; single arrow, neuronophagia directed at extracellular NM debris; double arrows, neuronophagia directed at a TH-immunonegative NM-laden neuron. Scale bar, 25 μm. **f** Top, Ipsilateral SNpc section from an AAV-hTyr-injected rat (4 m) immunostained with Iba1 (green) and astrocytic marker GFAP (purple), co-stained with blood vessel marker tomato lectin (red) and Hoechst (blue). Arrowheads, microglial cells carrying NM towards blood vessels. Arrow, perivascular NM. Scale bar, 25 μm. Bottom, Pearson correlation analysis between neuronophagia and perivascular NM. In **a**, **b** histograms, values are mean ± SEM. In **a**, *n* = 8 (0.5 m), *n* = 7 (1 m), *n* = 5 (2 m), *n* = 8 (4 m), *n* = 7 (12 m), *n* = 6 (24 m). In **b**, *n* = 6 (0.5 m), *n* = 5 (1 m), *n* = 5 (2 m), *n* = 7 (4 m), *n* = 6 (12 m), *n* = 6 (24 m). For Pearson correlation analyses each point represents the average value for the corresponding parameter at any given time post-AAV-hTyr injection (0.5, 1, 2, 4, 12, and 24 m). BF bright-field. In all panels, unstained NM appears as brown. Photomicrographs correspond to 5-μm-thick sections

Concomitant with SNpc DA neurodegeneration, abundant extracellular NM aggregates, released from dying neurons, were detected in the SNpc of AAV-hTyr-injected rats, as it is usually observed in aged and PD brains^39^ (Fig. 5a, c). Extracellular NM was associated with sustained microglial/macrophage activation (Fig. 5b, d), with NM fragments as well as entire NM-filled residual neurons being surrounded by, or contained within, activated microglia/macrophages (Fig. 5b–e). This phenomenon, known as neuronophagia, is commonly seen in post-mortem aged and PD brains^39^ (Fig. 5c) and is indicative of an active, ongoing neurodegenerative process^40^. Double immunofluorescence analyses revealed that neuronophagia was directed not only at extracellular NM debris but also, in some instances, at dysfunctional/dying NM-laden neurons that have lost their TH phenotype (Fig. 5e). In contrast, no neuronophagia was detected surrounding morphologically intact, apparently healthy, TH-positive NM-laden cells (Fig. 5e). Ultimately, and analogous to PD brains, NM-filled microglia from AAV-hTyr-injected rats appeared to migrate to blood vessels to exit the brain along with the NM pigment, as revealed by abundant perivascular NM in these animals (Fig. 5a, c, f). No neuroinflammatory changes were observed in the SNpc of AAV-EV-injected control rats nor in the contralateral (non-melanized) SNpc of AAV-hTyr-injected rats (Supplementary Figure 4).

DA oxidation to quinones is an essential event required for NM synthesis^41^ (Supplementary Figure 5). Because DA-derived quinones can be potentially toxic to DA neurons^42–45^, we next determined whether the neurodegenerative process observed in our NM-producing rats could be attributed to hTyr-mediated production of potentially toxic oxidized DA species. To address this issue, we developed a method to directly measure DA oxidation in AAV-hTyr-injected rats by ultra-performance liquid chromatography-tandem mass spectrometry (UPLC-MS/MS). Levels of oxidized DA were measured in both ipsilateral (i.e. melanized) and contralateral (i.e. non-melanized) ventral midbrains of AAV-hTyr-injected rats, at different times post-AAV injection: 1 m (i.e. before the occurrence of NM-linked neuronal dysfunction in these animals), 2 m (i.e. at the onset of neuronal dysfunction but before the occurrence of neurodegeneration) and 4 m (i.e. once neurodegeneration is established). No significant changes were detected between ipsilateral and contralateral ventral midbrain levels of oxidized DA at any of the times analyzed (Supplementary Figure 5), therefore arguing against a major significant contribution of oxidized DA species in the pathological changes observed in AAV-hTyr-injected rats. Further supporting this concept, we did not detect significant alterations in DA metabolism in these animals, either preceding or at the onset of neurodegeneration, as reflected by unchanged levels of DA and other DA metabolites in their ventral midbrains (Supplementary Figure 5).

Our results reveal that age-dependent NM production within SNpc DA neurons in AAV-hTyr-injected rats is associated with neuronal dysfunction and progressive nigrostriatal neurodegeneration, equivalent to that occurring in PD patients^46^, once a certain threshold of intracellular NM accumulation is reached. Remarkably, the intracellular NM levels above which AAV-hTyr-injected rats started exhibiting functional alterations (by 2 m) and overt neurodegeneration (by 4 m) were equivalent to those observed in ILBD subjects and PD patients (Fig. 2b, c), thus allowing a specific pathogenic threshold for intracellular NM accumulation to be defined (see dashed red lines in Fig. 2b, c). These results are consistent with intracellular NM levels setting a threshold for the initiation of PD-related neurodegeneration.

### PD-like inclusion formation in NM-producing rats

**Fig. 6:**
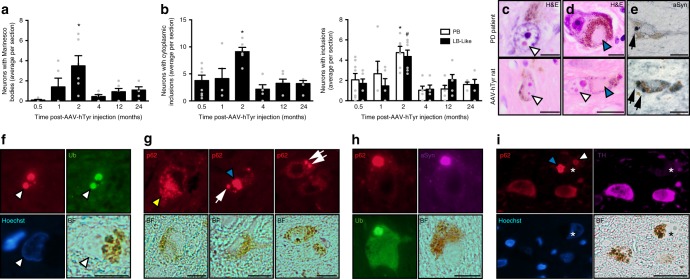
PD-type inclusion formation in AAV-hTyr-injected rats. **a** Quantification of NM-laden neurons with p62-positive intranuclear Marinesco bodies (MB) in ipsilateral SNpc from AAV-hTyr-injected rats. *p < 0.05, compared to 0.5 m (ANOVA on ranks; Dunn’s post-hoc test). **b** Quantification of NM-laden neurons with p62-positive total intracytoplasmic inclusion bodies (left) and pale bodies (PB) and/or Lewy body (LB)-like p62-positive inclusions (right) in ipsilateral SNpc from AAV-hTyr-injected rats. Left, *p < 0.05, compared to 0.5, 1, 4, 12, and 24 m (one-way ANOVA; Student–Newman–Keuls post-hoc test). Right, *p < 0.05, compared to PB at 0.5, 4, 12, and 24 m; #*p* < 0.05, compared to LB-like inclusions at 0.5, 1, 4, and 24 m (two-way ANOVA; Holm-Sidak post-hoc test). **c**–**e** Midbrain sections from PD post-mortem brains (top) and AAV-hTyr-injected rats (bottom) exhibiting MB (**c**, white arrowheads), PB (**d**, blue arrowheads) and LB-type aSyn-positive inclusions (**e**, arrows). In **c**, **d**, Hematoxylin-eosin (H&E) staining. In **e**, aSyn immunostaining (in blue). Scale bars, 12.5 μm. **f** Ipsilateral SNpc sections from AAV-hTyr-injected rats exhibiting NM-laden neurons with MB (white arrowhead) detected by immunofluorescence with p62 (red) and ubiquitin (Ub, green). Nuclei are stained with Hoechst (blue). Scale bar, 12.5 μm. **g** Ipsilateral SNpc sections from AAV-hTyr-injected rats exhibiting NM-laden neurons with p62-positive multipunctate cytoplasmic inclusions (yellow arrowhead), PB (blue arrowhead) or LB-like inclusions (arrows). Scale bar, 12.5 μm. **h** Ipsilateral SNpc section from an AAV-hTyr-injected rat exhibiting a NM-laden neuron with an intracytoplasmic LB-like inclusion immunopositive for p62 (red), aSyn (purple) and ubiquitin (Ub, green). Scale bar, 12.5 μm. **i** Ipsilateral SNpc section from an AAV-hTyr-injected rat immunostained for p62 (red) and TH (purple) in which a NM-laden neuron containing both a PB (blue arrowhead) and a MB (white arrowhead) exhibits decreased TH immunostaining (asterisk). Nuclei are stained with Hoechst (blue). Scale bar, 25 μm. In **a**, **b**, values are mean ± SEM. *n* = 8 (0.5 m), *n* = 5 (1 m), *n* = 6 (2 m), *n* = 5 (4 m), *n* = 6 (12 m), *n* = 5 (24 m). Ub ubiquitin, BF bright-field. In all panels, unstained NM appears as brown. Photomicrographs correspond to 5-μm-thick sections

In addition to neurodegeneration, ipsilateral NM-laden SNpc DA neurons from AAV-hTyr-injected rats also exhibited intracellular inclusion bodies typical of human post-mortem aged and PD brains, including nuclear Marinesco bodies (MB) and cytoplasmic PB and LB-like inclusions (Fig. 6). Inclusion body formation in these animals: (i) was restricted to NM-containing neurons, (ii) peaked at 2 m post-AAV-hTyr injection, thus coinciding with functional alterations and preceding neurodegeneration in these animals, and (iii) was substantially reduced by 4 m and onwards, once neurodegeneration was already established (Fig. 6a, b).

MB are spherical eosinophilic nuclear inclusions found in pigmented neurons of the human SNpc, the incidence of which increases with advancing age^47^. Similar to humans^47^, one or several MB were detected in the nucleus of pigmented SNpc neurons from AAV-hTyr-injected rats (Fig. 6c, d, f). In these animals, MB were intensely immunopositive for p62, a common component of neuropathological inclusions, and ubiquitin (Fig. 6f), as it has been previously reported in humans^47^. No MB were found in non-pigmented neurons from AAV-hTyr-injected rats. Concomitant with the occurrence of MB, NM-laden neurons from AAV-hTyr-injected rats also exhibited cytoplasmic PB and LB-like inclusions, often within the same cell, as is commonly seen in PD brains^47^ (Fig. 6c–i). No cytoplasmic inclusions were found in non-pigmented neurons from these animals. PB are identified as weakly or non-eosinophilic cytoplasmic areas of varied morphologies that displace NM, lacking the spherical and compact appearance of LB-like structures (Fig. 6d). As in PD, PB and LB-like inclusions from AAV-hTyr-injected rats were immunopositive for p62 and, in some instances, for aSyn (both full length and hyperphosphorylated) and ubiquitin (Fig. 6e, g–i and Supplementary Figure 6). This is consistent with previous studies in human PD brains reporting a selective, early, and consistent incorporation of p62 into both PB and LB, suggesting an important role for p62 in their formation, in contrast to a less consistent and less selective accumulation of ubiquitin within these cytoplasmic inclusions^47^. Cytoplasmic inclusions in AAV-hTyr-injected rats emerged within NM-filled areas, either overlapping with or displacing NM granules, and often appeared as early multiple punctate small aggregates immunopositive for p62 that seemed to progressively coalesce into more compacted LB-like spherical structures (Fig. 6g), as similarly reported in post-mortem PD brains^47^.

Taken together, our results show that intracellular NM accumulation is associated with the formation of PD-like neuronal inclusions in AAV-hTyr-injected rats. While the mechanism and significance of inclusion body formation in PD remain unknown, the temporal pattern of inclusion formation in AAV-hTyr-injected rats (i.e. preceding neurodegeneration and decreasing in parallel with neuronal death) suggests that inclusion-containing neurons are those that preferentially degenerate in these animals. In agreement with this, the number of neuronal inclusions in PD brains at advanced stages of the disease is much lower than that observed in early PD cases^14^, as it occurs in AAV-hTyr-injected rats. In addition, in both humans^48^ and AAV-hTyr-injected rats (Fig. 6i), inclusion-containing neurons often exhibit decreased intracellular TH levels, which reflects neuronal dysfunction at early stages of neurodegeneration^1^. Consistent with this, inclusion formation in AAV-hTyr-injected rats peaked at the onset of functional alterations (i.e. impaired DA release and hypokinesia) in these animals. Our results indicate that PD-type inclusion body formation reflects a pathologic process related or contributing to PD-linked neuronal dysfunction and degeneration.

### aSyn is dispensable for NM-linked PD pathology

**Fig. 7:**
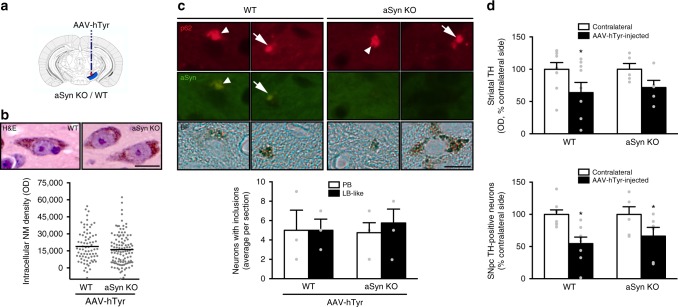
Dispensability of aSyn for PD-like inclusion formation and neurodegeneration linked to NM accumulation. **a** Schematic representation of the site of AAV-hTyr unilateral stereotaxic injection above the SNpc of aSyn knockout (KO) and wild-type (WT) mice. **b** Top, representative photomicrographs of NM-laden neurons in hematoxylin-eosin (H&E)-stained ipsilateral SNpc brain sections from AAV-hTyr-injected aSyn KO and WT mice at 6 m post-AAV injection. Scale bar, 12.5 μm. Bottom, quantification of intracellular NM optical density in ipsilateral SNpc DA neurons of AAV-hTyr-injected aSyn KO and WT mice. *p* = 0.879 (two-tailed *t*-test). **c** Top, ipsilateral SNpc sections from AAV-hTyr-injected aSyn KO and WT mice exhibiting NM-laden neurons with p62-positive (red) PB (arrowhead) and LB-like inclusions (arrow) at 2 m post-AAV injection. aSyn immunofluorescence is shown in green. Scale bar, 12.5 μm. Bottom, quantification of NM-laden neurons with p62-positive PB or LB-like inclusions in AAV-hTyr-injected aSyn KO and WT mice at 2 m post-AAV injection. *p* = 0.866 (two-way ANOVA). **d** Top, optical densitometry of striatal TH-positive fibers in AAV-hTyr-injected aSyn KO and WT mice at 6 m post-AAV injection. *p < 0.05, compared to respective contralateral (non-injected) side (two-way ANOVA; Student–Newman–Keuls post-hoc test). Bottom, stereological cell counts of SNpc TH-positive neurons in AAV-hTyr-injected aSyn KO and WT mice at 6 m post-AAV injection. *p < 0.05, compared to respective contralateral (non-injected) side (two-way ANOVA; Student–Newman–Keuls post-hoc test). In all panels, values are mean ± SEM. In **b**, *n* = 76 neurons from *n* = 4 mice (WT), *n* = 112 neurons from *n* = 4 mice (aSyn KO). In **c**, *n* = 3 (WT), *n* = 4 (aSyn KO) mice. In **d** (top), *n* = 9 (WT), *n* = 5 (aSyn KO) mice. In **d** (bottom), *n* = 8 (WT), *n* = 6 (aSyn KO) mice. BF bright-field. In all panels, unstained NM appears as brown. Photomicrographs correspond to 5-μm-thick sections

Given the presence of PD-like aSyn-positive inclusions in NM-producing rats and the potential pathogenic role attributed to aSyn aggregation in PD, we next assessed whether NM-linked PD-like pathology in AAV-hTyr-injected animals was dependent on aSyn. This question was addressed by inducing NM production with AAV-hTyr in the SNpc of aSyn-deficient mice (Fig. 7a). Because aSyn has been reported to be able to interact with tyrosinase^49^ and to modulate ultra-violet radiation-induced melanin synthesis in melanoma cells^50^, we first determined whether the lack of aSyn in aSyn-deficient mice could interfere with AAV-hTyr-induced production of NM in these animals. Ruling out this possibility, intracellular NM levels within AAV-hTyr-injected SNpc neurons were comparable between wild-type (WT) and aSyn-deficient mice at 6 m post-AAV-hTyr injection (Fig. 7b). By that time, WT and aSyn-deficient mice exhibited intracellular NM levels similar to the pathogenic threshold of intracellular NM accumulation defined above for rats and humans (Fig. 7b). We next assessed whether the absence of aSyn in aSyn-deficient mice may prevent or attenuate the formation of PD-like cytoplasmic inclusions within NM-laden neurons in AAV-hTyr-injected mice. At 2 m post-AAV-hTyr injections (i.e. at the peak of inclusion formation defined in AAV-hTyr-injected rats), NM-laden SNpc neurons from WT mice exhibited p62-immunopositive PB and LB-like inclusions at levels equivalent to those observed in AAV- hTyr-injected rats (Fig. 7c and Supplementary Figure 6). Unexpectedly, NM-laden SNpc neurons from AAV-hTyr-injected aSyn-deficient mice exhibited similar p62-immunopositive inclusions at levels comparable to those of WT animals (Fig. 7c), indicating that the lack aSyn did not prevent the formation of cytoplasmic inclusions. While in WT mice these inclusions contained aSyn, p62, and ubiquitin, in aSyn-deficient mice they were immunopositive for p62 and ubiquitin but lacked aSyn (Fig. 7c and Supplementary Figure 6). Finally, to determine whether aSyn actually contributed to NM-linked cell death in NM-producing animals, we compared nigrostriatal degeneration between AAV-hTyr-injected WT and aSyn-deficient mice. At 6 m post-AAV-hTyr injections, WT and aSyn-deficient mice exhibited comparable levels of striatal DA denervation and SNpc DA neurodegeneration (Fig. 7d), indicating that aSyn is not contributing to NM-linked cell death.

Overall, our results indicate that whereas NM accumulation above a pathogenic threshold is associated with the formation of PD-like aSyn-positive inclusions, aSyn is not actually required for neither inclusion formation nor nigrostriatal neurodegeneration in AAV-hTyr-injected animals.

### General proteostasis failure in NM-producing cells

The accumulation of p62-immunopositive inclusion bodies in NM-producing rats and the dispensability of aSyn for their formation suggests a general failure of cellular proteostasis, beyond aSyn, in NM-laden neurons. Indeed, p62-positive aggregates are typically formed following inhibition of autophagy or proteasome-mediated intracellular degradation^51^. Both autophagy and ubiquitin-proteasome (UPS) systems are defective within NM-laden, but not non-melanized, regions from post-mortem PD brains^52,53^. In neural cells, suppression of basal autophagy is sufficient to cause neurodegeneration, even in the absence of any disease-associated mutant proteins^54^.

**Fig. 8:**
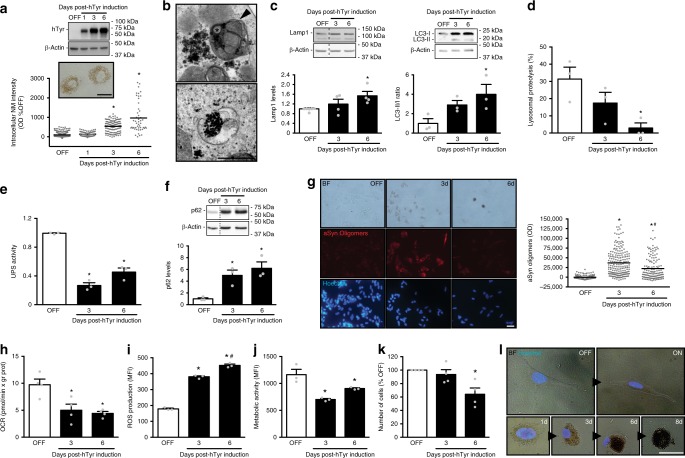
General proteostasis failure in NM-producing cells. **a** Top, Induction of hTyr expression in differentiated neuroblastoma SH-SY5Y cells before (OFF) and after (ON) treatment with doxycycline. Bottom, quantification of intracellular NM optical density post-hTyr induction. *p < 0.05, compared to OFF and ON 1d (ANOVA on ranks; Dunn’s post-hoc test). Scale bar, 12.5 μm. **b** Electron micrographs of NM granules in hTyr-expressing cells (6d post-hTyr induction). Arrowhead, double-membrane; asterisk, lipid droplet. Scale bar, 0.5 μm. **c** Immunoblot levels of Lamp1 (left) and LC3 (right) post-hTyr induction. **d** Lysosomal proteolysis post-hTyr induction. **e** Ubiquitin-proteasome (UPS) activity post-hTyr induction. **f** Immunoblot levels of p62 post-hTyr induction. **g** Representative images (left) and quantification (right) of aSyn oligomers (red) in hTyr-expressing cells as detected by aSyn-proximity ligation assay. Hoechst, nuclei (blue). BF bright-field. Scale bar, 25 μm. **h** Mitochondrial oxygen consumption rate (OCR) post-hTyr induction. **i** ROS production post-hTyr induction. MFI mean fluorescence intensity. **j** Cellular metabolic activity post-hTyr induction. **k** Number of surviving cells post-hTyr induction. **l** Representative bright-field photomicrographs with superimposed nuclear Hoechst fluorescent staining (blue) of hTyr-expressing cells before (OFF) and after (ON, 1–8d) hTyr induction. For clarity purposes, cell contour in weakly or no melanized cells are highlighted in white. Scale bar, 12.5 μm. In all panels, values are mean ± SEM. In **a**, **d**, **e** and **i**, *n* = 3 independent experiments. In **c**, **h**, **j** and **k**, *n* = 4 independent experiments. In **a** (bottom), *n* = 499 (OFF), *n* = 155 (1d), *n* = 111 (3d), and *n* = 48 (6d) cells. In **g**, *n* = 153 (OFF), *n* = 201 (3d), and *n* = 135 (6d) cells. Immunoblot densitometry was normalized to β-actin expression levels. BF bright-field, KDa kilodaltons. Unstained NM appears as brown. In **c**–**f**, **h**, **j**, *p < 0.05, compared to OFF (one-way ANOVA; Student–Newman–Keuls post-hoc test). In **g**, *p < 0.05, compared to OFF; #*p* < 0.05, compared to ON 3d (ANOVA on ranks; Dunn’s post-hoc test). In **i**, *p < 0.05, compared to OFF; #*p* < 0.05, compared to ON 3d (one-way ANOVA; Student–Newman–Keuls post-hoc test). In **k**, *p < 0.05, compared to OFF (ANOVA on ranks; Dunn’s post-hoc test)

To determine whether NM accumulation may indeed affect cellular proteostasis, additional experiments were performed in differentiated human catecholaminergic neuroblastoma SH-SY5Y cells inducible for hTyr expression. As in AAV-hTyr-injected rats, induction of hTyr expression in these cells resulted in a progressive intracellular production and accumulation of NM, until most of the cellular cytoplasm was occupied by 6 days (d) post-induction (Fig. 8a and Supplementary Figure 7). In humans, intracellular NM is known to continuously accumulate over a lifetime into autophagic structures^23^. In agreement with this, NM from both NM-producing rats and hTyr-expressing cells appeared enclosed within single-membrane-delimited mature lysosomes/autophagolysosomes (Supplementary Figure 8) or inside double-membrane-delimited autophagosomes (Fig. 8b), as assessed by electron microscopy examination. Consistent with the autophagic nature of NM granules, levels of the lysosomal structural marker Lamp1 and the autophagosome marker LC3-II/I increased in parallel with NM production in hTyr-expressing cells (Fig. 8c and Supplementary Figure 7) and Lamp1 was shown to co-localize with NM granules in AAV-hTyr-injected rodents (Supplementary Figure 8). The continuous buildup of NM within autophagic compartments was associated to a parallel decrease in lysosomal-mediated proteolysis in hTyr-expressing cells, as measured by intracellular protein degradation assay (Fig. 8d). In addition to impaired lysosomal proteolysis, UPS activity was also markedly reduced in NM-laden cells, as measured by quantification of chymotrypsin-like activity of the 20 S proteasome (Fig. 8e). Consistent with a failure in autophagy- and UPS-mediated proteolysis, NM-laden cells exhibited a marked accumulation of p62 protein by immunoblot (Fig. 8f and Supplementary Figure 7). In addition, NM-laden cells also exhibited extensive deposition of aSyn oligomers, as directly visualized by aSyn proximity ligation assay (PLA) (Fig. 8g), a method that has been previously used to detect aSyn oligomers in post-mortem PD brains^55^. Impairment of both autophagy and UPS is indicative of a general, late-stage proteostasis failure, in which cell function and survival become compromised^56^. For instance, mitochondrial quality control is tightly linked to the proteolytic cytosolic systems and if the latter are blocked, damaged mitochondria gradually accumulate^57^. Supporting this concept, NM-laden cells exhibited impaired mitochondrial respiration, as determined by respirometry with a Seahorse flux analyzer system (Fig. 8h), and increased production of reactive oxygen species (ROS), as assessed by flow cytometry with the green fluorescent dye CellROX (Fig. 8i). Concomitantly, these cells exhibited reduced metabolic activity, as revealed by a decreased capacity to reduce resazurin to red-fluorescent resorufin (Fig. 8j). These functional changes ultimately compromised cell viability, as NM-producing cells started to die by 6d post-hTyr induction (Fig. 8k). By that time, some NM-filled neurons exhibited a nucleus that was apparently displaced and extruded by NM while leaking their NM contents outside the cell as they degenerate (Fig. 8l). By 8d, most NM-laden cells have disappeared, with only some NM-filled cellular remains and extracellular residual NM persisting in the medium (Fig. 8l).

These results indicate that the continuous buildup of NM within autophagic compartments ultimately exhausts the autophagic capacity of the cell, leading to a general failure of proteostasis and subsequent dysfunction and degeneration of NM-laden cells.

### Modulation of NM levels and pathology in NM-producing rats

**Fig. 9:**
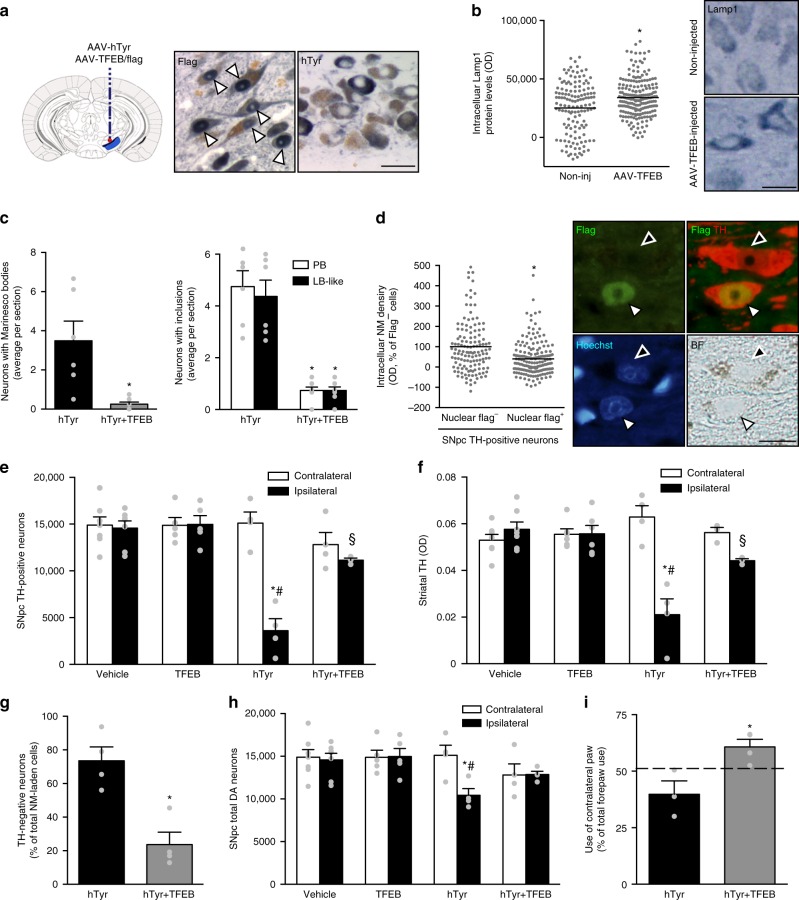
Therapeutic enhancement of lysosomal proteostasis in NM-producing rats. **a** Ipsilateral SNpc sections from rats co-injected with AAV-hTyr/flagged-AAV-TFEB immunostained with Flag (blue, left) or hTyr (blue, right) at 5 m post-AAV. NM, brown. Arrowheads, nuclear Flag/TFEB. Scale bar, 25 μm. **b** Lamp1 immunolabeling (blue) in ipsilateral SNpc of AAV-TFEB-injected rats (12 m post-AAV). *p < 0.05, compared to contralateral (non-injected) SNpc (Mann–Whitney rank sum test). Scale bar, 12.5 μm. **c** NM-laden neurons with p62-positive MB (left) and PB/LB-like inclusions (right) in ipsilateral SNpc from AAV-hTyr- and AAV-hTyr/TFEB-injected rats (2 m post-AAV). *p < 0.05, compared to AAV-hTyr-injected animals (left, Mann–Whitney rank sum test; right, two-way ANOVA, Student–Newman–Keuls post-hoc test). **d** Intracellular NM density in ipsilateral SNpc TH-positive neurons from rats co-injected with AAV-TFEB/AAV-hTyr (12 m post-AAV). White arrowhead, Flag/TFEB-positive nucleus; black arrowhead, Flag/TFEB-negative nucleus. Scale bar, 12.5 μm. *p < 0.05, compared to Flag/TFEB-negative neurons (Mann–Whitney rank sum test). **e** SNpc TH-positive neurons at 12 m post-AAV/vehicle injections. **f** Striatal TH-positive fibers at 12 m post-AAV/vehicle injections. In **e**, **f**, *p < 0.05, compared to respective contralateral side; #*p* < 0.05, compared to ipsilateral vehicle- and AAV-TFEB-injected animals; §*p* < 0.05, compared to ipsilateral AAV-hTyr-injected animals (two-way ANOVA; Student–Newman–Keuls post-hoc test). **g** TH downregulation within NM-laden neurons (12 m post-AAV). *p < 0.05, compared to AAV-hTyr-injected animals (two-tailed *t*-test). **h** Total SNpc DA neurons at 12 m post-AAV/vehicle injections. *p < 0.05, compared to respective contralateral side; #*p* < 0.05, compared to ipsilateral vehicle- and AAV-TFEB-injected animals (two-way ANOVA; Student–Newman–Keuls post-hoc test). **i** Contralateral forepaw use in AAV-hTyr- and AAV-hTyr/TFEB-injected rats (12 m post-AAV). *p < 0.05, compared to AAV-hTyr (only)-injected animals (two-tailed *t*-test). Dashed line indicates average contralateral forepaw use in control-injected rats. In all panels, values are mean ± SEM. In **b**, *n* = 138 neurons from *n* = 4 non-injected rats and *n* = 203 neurons from *n* = 5 AAV-TFEB-injected rats. In **c**, *n* = 6 (hTyr), *n* = 8 (hTyr + TFEB) rats. In **d**, *n* = 132 nuclear flag- neurons and *n* = 173 nuclear flag + neurons from *n* = 4 AAV-hTyr + TFEB-injected rats. In **e** and **h**, *n* = 7 (vehicle), *n* = 4 (TFEB), *n* = 5 (hTyr), *n* = 4 (hTyr + TFEB) rats. In **f**, *n* = 7 (vehicle), *n* = 6 (TFEB), *n* = 4 (hTyr), *n* = 3 (hTyr + TFEB) rats. In **g**, *n* = 4 animals per group. In **i**, *n* = 12 (control), *n* = 3 (hTyr), *n* = 4 (hTyr + TFEB) rats. BF bright-field. Photomicrographs correspond to 5-μm-thick sections

To corroborate whether proteostasis failure was indeed responsible for PD-like pathology linked to NM accumulation, we next assessed the effects of enhancing proteostasis with transcription factor EB (TFEB) in NM-producing rats. TFEB is a master regulator of autophagy that upon activation translocates into the nucleus and induces the biogenesis of lysosomes and autophagosomes, boosts autophagic cellular clearance, promotes lysosomal exocytosis, and modulates general proteostasis^58–62^. By doing so, TFEB overexpression has been shown to protect against pathological substrate accumulation and cell death in several rodent models of lysosomal storage disorders and neurodegenerative diseases^63^. To assess the effects of TFEB on NM-producing rats, both AAV-TFEB and AAV-hTyr were co-injected into the rat SNpc, with additional control groups of animals receiving equivalent amounts of either vehicle, AAV-hTyr or AAV-TFEB, separately. Co-injection of AAV-TFEB and AAV-hTyr resulted in a marked nuclear expression of TFEB within SNpc neurons, without interfering with hTyr expression or NM production in these cells at 5 m post-AAV injection (Fig. 9a). In agreement with the well-established role of TFEB at inducing lysosomal biogenesis, SNpc DA neurons from AAV-TFEB-injected animals exhibited increased levels of Lamp1, up to 1 year post-AAV injection (Fig. 9b). In hTyr-expressing cells, TFEB overexpression was also confirmed to induce lysosomal exocytosis (Supplementary Figure 8), as part of its known effects at promoting cellular clearance^60,62^. In NM-producing rats, TFEB overexpression markedly reduced inclusion body formation, both in the nucleus (i.e. MB) and the cytosol (i.e. PB and LB-like) of NM-laden neurons, compared to AAV-hTyr (only)-injected animals (Fig. 9c). Remarkably, intracellular NM density was also markedly reduced by TFEB within neurons displaying overtly activated (i.e. nuclear) TFEB expression (Fig. 9d). TFEB-induced reductions in intracellular NM and inclusion body formation were associated, in turn, with a marked attenuation of NM-linked neurodegenerative changes in hTyr/TFEB-expressing rats, including attenuated loss of SNpc TH-positive cells, decreased nigrostriatal denervation, reduced TH downregulation, and diminished total SNpc DA neuronal death, all of which was associated to a marked improvement of contralateral forepaw hypokinesia in these animals (Fig. 9e–i). Our results indicate that NM-linked impaired proteostasis plays a pivotal role in the initiation of PD-like pathology in AAV-hTyr-injected rats.

## Discussion

In the present work, we have generated the first experimental in vivo model of age-dependent production and intracellular accumulation of human-like NM within PD-vulnerable SNpc DA neurons, up to levels reached in elderly humans. While the aim of generating such a model was to assess the consequences of progressive intracellular NM accumulation, our results may also shed light on the actual mechanisms of NM synthesis in humans, which are currently poorly understood. While it is generally accepted that NM is formed by non-enzymatic DA autoxidation^41^, in contrast to the well-known enzymatic synthesis of peripheral melanins^18^, the striking resemblance between human NM and NM from AAV-hTyr-injected rats raises the question of a potential enzymatic contribution to NM synthesis, either by tyrosinase itself, which is present at low levels in human SNpc, or by alternative tyrosinase-related enzymes. Further supporting a potential enzymatic involvement in NM synthesis, it has been reported that increased levels of DA and oxidized DA in mice and rats, either with chronic L-DOPA treatment^44,64^ or TH overexpression^45^, is apparently not sufficient by itself to produce NM in these animals, as might be expected if NM represents a mere process of autoxidized DA. In contrast, in the present study, conspicuous NM production was obtained solely by overexpressing hTyr in the rodent brain. In addition, our observation of tyrosinase expression being normally present at low levels in the human brain further supports the possibility of a potential enzymatic contribution to NM synthesis. The latter results concur with previous reports having detected the presence of brain tyrosinase in humans at the level of mRNA, protein, and enzymatic activity^20–22^, as well as with results from three different microarray experiments in human brain tissue (Entrez_id: 7299; Allen Brain Institute), non-human primate brain tissue (Entrez_id: 705792; NIH Blueprint NHP Atlas), and developing human prenatal brain tissue (Entrez_id: 7299, BrainSpan Atlas of the developing human brain) consistently detecting the presence of tyrosinase transcripts at low levels. In rodents, tyrosinase expression has been previously detected in mouse SN by in situ hybridization (Allen Brain Atlas data portal) and qPCR^65^. However, other studies have failed to detect tyrosinase expression in the human brain^66,67^. The apparently conflicting results regarding the presence or absence of tyrosinase in the human brain may be attributed to the low levels at which tyrosinase may be actually expressed in the brain and thus the requirement of adequate methodological approaches for its detection. For instance, brain tyrosinase has not been detected in humans when using immunohistochemistry or western blot (WB) from total protein homogenates^66,67^, two techniques unable to detect such low levels of protein expression at which tyrosinase may be present in the brain. In contrast, tyrosinase expression has been systematically detected in the human brain when using either RT-PCR/qPCR, in situ hybridization or WB with tyrosinase-immunoprecipitated protein samples^20–22^ (see also Supplementary Figure 1). Further studies are required to determine in a definite manner a potential contribution of tyrosinase or other enzymes in NM synthesis.

The functional significance of NM production remains speculative. Until recently, NM was considered merely as a waste product of cellular metabolism that was devoid of any physiological function. However, increasing evidence indicates that NM can interact with the cellular environment and influence cell function and survival. For instance, similar to peripheral melanins, NM possesses antioxidant and free radical scavenging properties, which might be important for catecholaminergic neurons, as these cells endure a particularly high oxidative burden^68^. In this context, it has been proposed that the synthesis of NM may represent an antioxidant mechanism to remove excessive cytosolic DA-derived quinones and semiquinones, thereby blocking their potential toxicity^68^. Supporting this concept, hTyr-induced oxidized DA species did not significantly increase in hTyr-injected rats, suggesting that the continuous conversion of these species into NM may be preventing their potentially toxic accumulation in these animals. Moreover, NM is able to bind and sequester potentially toxic metals, in particular redox-active ferric ions, as well as environmental parkinsonian toxins, such as 1-methyl-4-phenylpyridinium (MPP^+^, the active metabolite of MPTP) or the pesticide paraquat^69^. Despite a putative beneficial role of NM synthesis, our results suggest that the progressive buildup of intracellular NM may ultimately interfere with normal cell function and trigger PD-like neuronal dysfunction/degeneration when reaching a pathogenic threshold of accumulation, regardless of whether the initial synthesis of NM is a protective or inert process.

Neuronal dysfunction/degeneration in NM-producing AAV-hTyr-injected rats could potentially result from DA-mediated neurotoxicity by increased hTyr-mediated production of potentially toxic oxidized DA species. However, as discussed above, the continuous formation of NM in AAV-hTyr-injected rats seems to prevent the significant accumulation of oxidized DA species in these animals. Further arguing against a major pathogenic role for oxidized DA species in AAV-hTyr-injected rodents, it has been recently reported by two independent groups^44,45^ that DA-mediated toxicity in rodents: (i) is only observed in animals displaying additional PD-related alterations, such as DJ-1 deficiency^44^ or overexpression of PD-linked A53T mutant aSyn^45^, but not in regular WT animals^44,45^, in contrast to our results in AAV-hTyr-injected WT rodents; (ii) is dependent on aSyn^44,45^, in contrast to the dispensability of aSyn that we reported for AAV-hTyr-mediated toxicity. In this context, the continuous accumulation of NM over a lifetime into undegraded or partly degraded autophagic structures, either in an attempt by the cell to degrade this pigment or to isolate it from the rest of the cell to prevent its potential pathological interaction with cytosolic cellular components, may ultimately exhaust the vesicular storage capacity of the cell, interfere with lysosomal proteases and other degradative pathways, impair intracellular vesicular trafficking and alter endocytic/secretory tasks^17,23^. Consistent with such a scenario, here we found that NM accumulation was associated to a general impairment of cellular proteostasis accompanied by the formation of PD-like intracellular inclusions, neuronal dysfunction, and neurodegeneration. Supporting a pivotal detrimental role for impaired proteostasis in NM-laden cells, enhancement of lysosomal-mediated proteolysis by TFEB overexpression in NM-producing rats reduced intracellular NM density, attenuated PD-like inclusion formation, prevented nigrostriatal neurodegeneration, and reversed motor impairment.

The results presented here could explain the major pathologic features of PD, including: (i) the selective vulnerability of specific neuronal groups (i.e. those containing NM); (ii) the correlation of PD with age (as NM accumulates with age); (iii) the reported cellular alterations linked to PD, such as mitochondrial dysfunction, increased ROS production, autophagy/UPS defects, inclusion body formation, and neuroinflammation, all of which have been proposed as potential primary pathogenic events in PD but, in light of our results, may in fact occur concomitantly secondarily to intracellular MN accumulation; (iv) the uniqueness of PD to humans, since only humans accumulate such high levels of intracellular NM^16^. However, while NM is present in the brain of all humans, only some individuals actually develop PD (1 to up ~5% of the population over 60, increasing with age)^70^. Based on our findings, PD will appear in those subjects that have reached earlier the pathogenic threshold of intracellular NM accumulation. Supporting this concept, we found that intracellular NM levels appear indeed above this pathogenic threshold in the brains of PD patients and pre-symptomatic PD subjects (i.e. ILBD) while in the brains of elderly, aged-matched healthy individuals intracellular NM levels were maintained below this pathogenic threshold. In PD patients, the intracellular buildup of NM might be accelerated, thereby reaching earlier the pathogenic threshold of intracellular NM accumulation. This could occur, for instance: (i) by an abnormal upregulation/increased activity of hTyr or other putative NM-producing enzymes, such as tyrosinase-related protein 2 (i.e. dopachrome tautomerase), the expression of which we found significantly increased in induced pluripotent stem cell (iPSC)-derived DA neurons from PD patients;^71^ or (ii) by increased cytosolic DA levels, which may convert to NM, as a consequence of defects in VMAT2-dependent DA encapsulation within synaptic vesicles, as it has been shown in early PD cases^26^.

Based on our results, strategies to maintain or decrease intracellular NM levels below their pathogenic threshold may provide unprecedented therapeutic opportunities to prevent, halt, or delay neuronal dysfunction and degeneration linked to PD. Given the putative protective role of NM synthesis (see above), therapeutic strategies aimed at maintaining or decreasing intracellular NM levels below their pathogenic threshold should be preferentially targeted at preventing NM accumulation once NM has already been produced, for instance by promoting its elimination as reported here with TFEB. In contrast, the inhibition of NM synthesis itself may prove instead detrimental. For instance, a rare loss-of-function mutation in hTyr linked to albinism has been recently associated to an increased risk for PD^72^, which has been attributed to the possible inability of this hTyr variant to synthesize NM and the subsequent accumulation of potentially toxic DA-derived species that cannot be detoxified by its conversion into NM. In this context, it would be very important for any therapeutic strategy aimed at modulating NM levels to tightly regulate NM levels above its protective threshold and below its pathogenic threshold revealed here.

Overall, with the introduction into experimental in vivo research of a factor so intimately linked to PD such as NM, which has been so-far neglected in PD animal modeling, the results presented here may open new research avenues and lead to a paradigm shift in the field of PD and, in a broader sense, brain aging.

## Methods

### Animals

Adult male Sprague–Dawley rats (Charles River), 225–250 g at the time of surgery, were housed two to three per cage with ad libitum access to food and water during a 12 h (h) light/dark cycle. All the experimental and surgical procedures were conducted in strict accordance with the European (Directive 2010/63/UE) and Spanish laws and regulations (Real Decreto 53/2013; Generalitat de Catalunya Decret 214/97) on the protection of animals used for experimental and other scientific purposes, and approved by the Vall d’Hebron Research Institute (VHIR) Ethical Experimentation Committee. Rats were randomly distributed into the different experimental groups and control and experimental groups were processed at once to minimize bias. When appropriate, additional sporadic experiments were performed in aSyn-deficient^73^ and wild-type C57BL/6 mice, as indicated.

### Viral vector production

Recombinant AAV vector serotype 2/1 expressing the human* tyrosinase* cDNA driven by the CMV promoter (AAV-hTyr) and the corresponding control empty vector (AAV-EV) were produced at the Viral Vector Production Unit of the Autonomous University of Barcelona (UPV-UAB, Spain). Briefly, rAAV vectors were produced by triple transfection of 2 × 10^8^ HEK293 cells with 250 µg of pAAV, 250 µg of pRepCap, and 500 µg of pXX6 plasmid mixed with polyethylenimine (PEI; branched, MW 25,000; Sigma-Aldrich). The UPV-UAB generated a pAAV plasmid containing the ITRs of the AAV2 genome, a multicloning site to facilitate cloning of expression cassettes, and ampicillin resistance gene for selection. 48 h after transfection, cells were harvested by centrifugation (200 × *g*, 10 min); resuspended in 30 ml of 20 mM NaCl, 2 mM MgCl_2_, and 50 mM Tris-HCl (pH 8.5), and lysed by three freeze–thaw cycles. Cell lysate was clarified by centrifugation (2000 × *g*, 10 min) and the rAAV particles were purified from the supernatant by iodixanol gradient as previously described (Zolotukhin et al., 1999 Gene Therapy). The clarified lysate was treated with 50 U/ml of Benzonase (Novagen; 1 h, 37 °C) and centrifuged (3000 × *g*, 20 min). The vector-containing supernatant was collected and adjusted to 200 mM NaCl using a 5-M stock solution. To precipitate the virus from the clarified cell lysate, polyethylene glycol (PEG8000; Sigma-Aldrich) was added to a final concentration of 8% and the mixture was incubated (3 h, 4 °C) and centrifuged (8000 × *g*, 15 min). The AAV-containing pellets were resuspended in 20 mM NaCl, 2 mM MgCl_2_, and 50 mM Tris-HCl (pH 8.5) and incubated for 48 h at 4 °C. The rAAV titration method used was based on the quantitation of encapsidated DNA with the fluorescent dye PicoGreen® as previously described^74^, and the following vector concentrations were obtained: AAV-hTyr: 1.7 × 10^13^ gc/mL (batch a), 2.43 × 10^12^ gc/mL (batch b); AAV-EV: 2.48 × 10^13^ gc/mL.

AAV serotype 2/9 containing the murine *Tfeb* cDNA fused to 3 Flag epitopes under control of the cytomegalovirus (CMV) promoter (AAV-TFEB; concentration: 3 × 10^12^ gc/mL) was provided by TIGEM AAV Vector Core Facility (Italy)^59^.

### Surgical procedures

All surgical procedures on rodents were performed with the animals placed in a stereotaxic frame under general anesthesia using isofluorane (5% for the induction phase and 2% for the maintenance phase) (Baxter). Vector solutions were injected using a 10 μL Hamilton syringe fitted with a glass capillary (Hamilton model Cat#701). Animals received 2 μL (rats) or 1 μl (mice) of either AAV-hTyr, AAV-EV, vehicle or, in the experiments involving TFEB, a 1:1 mixture of AAV-hTyr/AAV-TFEB, AAV-hTyr/vehicle, or AAV-TFEB/vehicle. In both rats and mice, infusion was performed at a rate of 0.4 μL/min and the needle was left in place for an additional 4 min period before it was slowly retracted. Injection was carried out unilaterally on the right side of the brain at the following coordinates (flat skull position), right above the substantia nigra pars compacta (SNpc): antero-posterior: −5.2 mm (rats), −2.9 mm (mice); medio-lateral: −2 mm (rats), −1.3 mm (mice); dorso-ventral: −7.6 mm (rats), −4.2 mm (mice) below dural surface, calculated relative to bregma according to the stereotaxic atlas of Paxinos and Watson^75^.

### Transduction efficiency

AAV-hTyr transduction efficiency was determined at 2–4 weeks post-AAV injection (*n* = 6–8 rats) either by: (i) assessing hTyr expression within SNpc tyrosine hydroxylase (TH)-positive cells by double immunofluorescence with antibodies against TH and hTyr in six coronal midbrain sections through the entire SNpc, using the Cell Counter plugin on ImageJ software (Rasband, W.S., ImageJ, U. S. National Institutes of Health, Bethesda, Maryland, USA, 1997-2016); (ii) by counting the number of NM-positive neurons versus the total number of SNpc TH-positive neurons, as described below.

### Brain processing for histological analyses

Animals were deeply anesthetized with sodium pentobarbital (50 mg/kg, i.p.) and then perfused through the left ventricle with saline [0.9% (wt/vol)] at room temperature (RT), followed by ice-cold formaldehyde solution 4% phosphate buffered for histology (Panreac). The brains were removed and post-fixed for 24 h in the same fixative and subsequently processed for paraffin embedding following standard procedures or cryoprotected for 24–48 h in 30% sucrose at 4 °C and frozen. Sectioning was performed with a sliding microtome (Leica, Germany) at 5-µm-thickness for paraffin samples or in a cryostat at 20- or 30-µm-thickness for frozen samples (Leica, Germany).

### Immunohistochemistry

Deparaffinized rodent brain sections were quenched for 10 min in 3% H_2_O_2_-10% (vol/vol) methanol. Antigen retrieval in paraffin sections was performed with a 10-mM citric acid solution at pH 6.0 in a microwave for 20 min. Sections were rinsed 3 times in 0.1 M Tris buffered saline (TBS) between each incubation period. Blocking for 1 h with 5% (vol/vol) normal goat serum (NGS, Vector Laboratories) was followed by incubation with the appropriate primary antibody at 4 °C for 48 h in 2% (vol/vol) serum and with the corresponding biotinylated antibody (Vector Laboratories). Sections were visualized by incubation with avidin–biotin–peroxidase complex (Ultrasensitive and Immunopure ABC Peroxidase staining kits for the striatum and for the SNpc, respectively; Thermo Fisher Scientific), using the VectorSG Peroxidase Substrate Kit (Vector Laboratories) as a chromogen, and then mounted and coverslipped with DPX mounting medium (Sigma-Aldrich). Bright-field section images were examined using: (i) Zeiss AX10 LabA1 microscope coupled to an AxioCam ERc5s camera; (ii) Zeiss Imager.D1 microscope coupled to an AxioCam MRc camera; (iii) Zeiss Stemi 2000-C magnifying glass coupled to an AxioCam ERc5s camera. Images were processed with ZEN 2011 software (Zeiss, Germany). For immunofluorescence, a similar protocol was used without the quenching step. Preincubation was performed with 5% (vol/vol) NGS and 0.1% (vol/vol) Triton X-100 (Sigma-Aldrich) in phosphate buffered saline (PBS) solution. Corresponding primary antibodies were incubated together overnight at 4 °C in 2% (vol/vol) serum and adequate Alexa 488, 594, and 647-conjugated secondary antibodies (1:1000, Thermo Fisher Scientific) were incubated simultaneously for 1 h at RT in 2% (vol/vol) serum. Nuclei were stained with Hoechst 33342 (1:2000, Thermo Fisher Scientific) in 1× PBS for 10 min. Sections were coverslipped using the DakoCytomation Fluorescent Mounting Medium (Dako). Immunofluorescence section images were examined using: (i) an Olympus FSX100 microscope with a DP72 incorporated camera and processed with the FSX-BSW software (Olympus, Germany), and in an Olympus BX61 microscope with a DP72 camera and processed with the Cell Sens Entry v1.8 software (Olympus, Germany).

Primary antibodies and dilution factors were as follows: mouse anti-DAT (1:500, Novus Biologicals, Cat#NBP2-22164); guinea pig anti-VMAT2 (1:5000, Progen, Cat#16085); rabbit anti-TH (1:40000 for SNpc and 1:3500 for striatum immunohistochemistry in paraffin sections; 1:2000 for SNpc and 1:5000 for striatum immunohistochemistry in free-floating sections; 1:1000 for immunofluorescence, Calbiochem, Cat#657012); mouse anti-TH (1:1000, Merck Millipore, Cat#MAB5280); mouse anti-tyrosinase (1:500, Thermo Fisher Scientific, Cat#MS-800-P1); mouse anti-FLAG (1:1000, Sigma-Aldrich, Cat#F3165); rabbit anti-GAD65 + GAD67 (1:750, Abcam, Cat#ab49832); mouse anti-GFAP (1:1000, Sigma-Aldrich, Cat#G3893); rabbit anti-Iba1 (1:1000, Wako, Cat#019-19741); mouse anti-CD68 (1:100, Serotec, Cat#MCA341R); DyLight 594 Lectin (1:500, Vector Laboratories, Cat#DL-1177); guinea pig anti-p62 (1:1000, Progen, Cat#GP62-C); rabbit anti-Ubiquitin (1:500, Dako, Cat#Z0458); mouse anti-aSyn (1:1000, BD Biosciences, Cat#610786); mouse anti phosphorylated(Ser129)-aSyn (1:1000, Wako, Cat#015-25191); rabbit anti-Lamp1 (1:250, GeneTex, Cat#GTX19294); rat anti-Lamp1 (1:4000, Santa Cruz, Cat#sc-19992).

### Masson-Fontana and hematoxylin-eosin stainings

Staining of NM granules in 5-µm-thick paraffin-embedded rat brain sections was performed using the Masson-Fontana Staining Kit (DiaPath); this procedure is based on the ability of NM to chelate metals by reducing silver nitrate to a visible metallic state. Briefly, paraffin tissue sections were dewaxed and rehydrated by heating at 60 °C for 10 min, followed by xylene (5 min, 3 times) and ethanol serial washes (100-95-70%-H_2_O, 5 min each). Staining was performed by incubating the sections with ammoniac solution for 40 min at 56 °C, followed by sodium thiosulphate for 2 min and a final counterstain with Kernechtrot for 7 min. Between each step, samples were rinsed in distilled water. Standard hematoxylin-eosin (H&E) staining was performed in 5-µm-thick paraffin-embedded SNpc section for each animal. In these sections, SNpc DA neurons were identified by the visualization of unstained NM pigment.

### Magnetic resonance imaging

^1^H-Magnetic resonance imaging (MRI) studies in rats were performed in a 7T Bruker BioSpec 70/30 USR (Bruker BioSpin GmbH, Karlsruhe, Germany) equipped with a mini-imaging gradient set (400 mT/m), a linearly polarized transmit volume coil (72 mm inner diameter) and a dedicated brain surface coil as receiver. All MRI data were acquired and processed on a Linux computer using Paravision 5.1 software (Bruker BioSpin GmbH, Karlsruhe, Germany). Fixed rat brains were placed in a 15-ml Falcon tube and embedded in 2% agarose to diminish susceptibility artefacts. Low-resolution T2-weighted fast spin-echo images were initially obtained in axial, sagittal, and coronal planes to be used as reference scout images. Imaging parameters for these images were: effective echo time (TEeff) = 36 ms; repetition time (TR) = 3 s; echo train length (ETL) = 8; field of view (FOV) = 6 × 6 cm^2^; matrix size (MTX) = 128 × 128; slice thickness (ST) = 2 mm; gap between slices (gap) = 0.5 mm; number of slices (NS) = 25 -axial, 10-sagittal, 11 -coronal; number of averages (NA) = 1. High-resolution T1-weighted spin-echo images were acquired afterwards in the axial and coronal planes containing the region of interest with the following parameters: TE = 9 ms; TR = 500 ms; NA = 128; NS = 20 (axial) and 7 (coronal); FOV = 1.92 × 1.92 cm^2^; MTX = 128 × 128 (axial) and 192 × 128 (coronal); ST = 0.25 mm (axial) and 0.35 (coronal); thus resulting in a spatial resolution of 150 × 150 × 250 µm^3^ (axial) and 100 × 150 × 350 µm^3^ (coronal). The acquisition time was 2 h and 16 min for each high-resolution image.

### Intracellular NM quantifications

An average of 165 neurons (94 in mice) were analyzed at each time-point post-AAV-hTyr injection, i.e. 0.5, 1, 2, 4, 12, and 24 months (m), corresponding to 5–7 different animals per time-point. For the experiments related to TFEB, an average of 108 neurons were analyzed at 12 m post-AAV injections, corresponding to 3–4 different animals per experimental group. In all cases, a representative 5-µm-thick paraffin-embedded H&E-stained rat midbrain section was selected for each animal. In each section, all NM-positive neurons were examined at an objective magnification of ×63. Images covering all the SNpc region were taken with a Zeiss Imager. D1 microscope was coupled to an Axiocam MRc camera. Quantification of the intracellular density of NM pigment was achieved by means of optical densitometry using ImageJ software (NIH, USA). The pixel brightness values for all individual NM-positive cells (excluding the nucleus) in all acquired images were measured and corrected for non-specific background staining by subtracting values obtained from the neuropil in the same images. In animals co-injected with AAV-hTyr and AAV-TFEB, additional quantifications of intracellular NM density were performed as above in bright-field photomicrographs at ×63 magnification, taking into account whether NM-laden cells exhibited flagged TFEB-positive or TFEB-negative nuclei. All quantifications were performed by an investigator blinded to the experimental groups. For NM quantifications in post-mortem human samples and cultured cells, see details below. Of note, because intracellular NM density in both humans and rodents was measured as the intensity of the NM signal normalized to the respective neuronal surface (excluding the nucleus), these values can be compared between species.

### Stereological cell counting

Assessment of the total number of SNpc TH-positive neurons, the number of SNpc NM-laden neurons and the total number of DA neurons in the SNpc was performed according to the fractionator principle, using the MBF Bioscience StereoInvestigator 11 (64 bits) Software (Micro Brightfield). Serial 5-µm-thick paraffin-embedded sections covering the entire SNpc were included in the counting procedure (every 17th section in rats and every sixth section in mice, for a total of 6–8 sections analyzed/animal). The following sampling parameters were used: (i) a fixed counting frame with a width and length of 50 μm; (ii) a sampling grid size of 100 × 75 μm in rats and of 125 × 100 μm in mice. The counting frames were placed randomly by the software at the intersections of the grid within the outlined structure of interest. The cells in one brain side, contra- or ipsilateral to the injection site, were counted following the unbiased sampling rule using a ×100 lens and included in the measurement when they came into focus within the dissector. A coefficient of error of <0.10 was accepted. Data for the total numbers of TH-positive neurons and NM-containing neurons in the SNpc are expressed as the absolute numbers in the non-injected contralateral side and in the AAV-injected ipsilateral side. The total number of SNpc DA neurons was calculated by considering all TH^+^NM^+^, TH^−^NM^+^, and TH^+^NM^−^ neurons. The percentage of TH down-regulation was calculated by considering the total number of TH^+^NM^+^ and the total number of TH^−^NM^+^ with respect to the total number of neurons containing NM in the different experimental groups. AAV-hTyr-injected rats were analyzed at different time-points after injection: 0.5 m (*n* = 8), 1 m (*n* = 7), 2 m (*n* = 5), 4 m (*n* = 8), 12 m (*n* = 7), and 24 m (*n* = 6). AAV-hTyr (*n* = 5), AAV-TFEB (*n* = 4), AAV-hTyr + TFEB (*n* = 4), and vehicle-injected rats (*n* = 7) were analyzed at 12 m post-injection. AAV-EV-injected rats were analyzed at 4 m (*n* = 7), 12 m (*n* = 4), and 24 m (*n* = 3) post-injection. Vehicle-injected rats were analyzed at 4 and 12 m post-surgery (*n* = 8 animals/group). AAV-hTyr-injected mice were analyzed at 6 m post-AAV injections: WT (*n* = 8), aSyn KO (*n* = 6). All quantifications were performed by an investigator blinded to the experimental groups.

### Quantification of neuropathological parameters

The absolute number of extracellular NM aggregates was estimated within the same sections in which SNpc TH-positive stereological cell counts were performed (i.e. serial 5-µm-thick paraffin-embedded sections covering the entire SNpc, taking every 17th section in rats and every 6th section in mice, for a total of 6–8 sections analyzed/animal). The number of neuronophagic events were assessed in serial H&E-stained 5-µm-thick paraffin-embedded sections covering the entire SNpc. The number of p62-immunopositive Marinesco bodies, PB, and Lewy body-like inclusions was counted from SNpc sections fluorescently immunostained with guinea pig anti-p62 (1:1000, Progen), rabbit anti-ubiquitin (1:500, Dako), and mouse anti-aSyn (1:1000, BD Biosciences). The total number of p62-positive inclusions falling into each category was counted from images covering the whole SNpc region in each section. Quantifications were performed in AAV-hTyr-injected at different time-points post-AAV-hTyr injection: 0.5 m (*n* = 8), 1 m (*n* = 5), 2 m (*n* = 6), 4 m (*n* = 5), 12 m (*n* = 6), and 24 m (*n* = 5). AAV-hTyr-injected mice were analyzed at 2 m post-AAV injections: WT (*n* = 3), aSyn KO (*n* = 4). AAV-hTyr (*n* = 5), AAV-TFEB (*n* = 4), AAV-hTyr + TFEB (*n* = 4), and vehicle-injected rats (*n* = 7) were analyzed at 12 m post-injection. All quantifications were performed by an investigator blinded to the experimental groups.

### Optical densitometry analyses

The density of TH-positive fibers in the striatum was measured by densitometry in serial coronal sections covering the whole region (10 sections/animal). TH-immunostained 5-µm-thick paraffin-embedded sections were scanned with an Epson Perfection v750 Pro scanner and the resulting images were quantified using Sigma Scan Pro 5 software (Systat Software Inc, USA). Striatal densitometry values were corrected for non-specific background staining by subtracting densitometric values obtained from the cortex. Data are expressed as the percentage of the densitometric value of the equivalent anatomical area from the non-injected contralateral side of the same animal. For the AAV-hTyr groups, animals were analyzed at different time-points after AAV-hTyr injection: 0.5 m (*n* = 8), 1 m (*n* = 7), 2 m (*n* = 5), 4 m (*n* = 8), 12 m (*n* = 7), and 24 m (*n* = 6). For the AAV-hTyr/TFEB-related experiments, animals were analyzed at 12 m post-injection: AAV-hTyr (*n* = 4), AAV-TFEB (*n* = 6), AAV-hTyr + TFEB (*n* = 3), and vehicle-injected rats (*n* = 7). For control experiments, animals were analyzed at different time-points after injection: AAV-EV-injected rats at 4 m (*n* = 7), 12 m (*n* = 4), and 24 m (*n* = 3); vehicle-injected rats at 4 and 12 m (*n* = 8 animals per group). AAV-hTyr-injected mice were analyzed at 6 m post-AAV injections: WT (*n* = 9), aSyn KO (*n* = 5). All quantifications were performed by an investigator blinded to the experimental groups.

The density of GAD65 + GAD67-positive fibers in the superior colliculus was measured by densitometry in selected coronal sections around Bregma −5.64 mm. GAD65 + GAD67-immunostained 5-µm-thick paraffin-embedded sections were scanned as previously described and densitometry values were corrected for non-specific background staining by subtracting densitometric values obtained from the adjacent ventral parenquima. Data are expressed as the percentage of the densitometric value of the equivalent anatomical area from the non-injected contralateral side of the same animal. Animals were analyzed at different time-points: (i) naive rats: 4 m (*n* = 4) and 12 m (*n* = 5); (ii) vehicle-injected rats: 4 m (*n* = 6) and 12 m (*n* = 5); (iii) AAV-EV-injected rats: 12–24 m (*n* = 4); (iv) AAV-hTyr-injected rats: 4 m (*n* = 3) and 12–24 m (*n* = 7).

Lamp1 protein expression levels in SNpc neurons from AAV-TFEB-injected rats (*n* = 5 animals) were measured by intracellular optical densitometry using ImageJ software. Five-µm-thick paraffin-embedded SNpc sections were immunostained for Lamp1 and 200 cells were randomly selected for analysis in the AAV-TFEB-injected ipsilateral side and the non-injected contralateral side of the same animal. All quantifications were performed by an investigator blinded to the experimental groups.

### Cylinder behavioral test

Rats were tested for left and right forepaw use with the cylinder test 1 week before surgery (to establish the basal conditions for each animal) and at different times post-AAV-hTyr injection thereafter (0.5, 1, 2, 3, 6, 12, and 24 m). Rats that presented an asymmetric usage of the right-left forepaws at basal examination were excluded from the analysis. At the beginning of the experiment, the number of animals for each condition was: *n* = 29 (AAV-hTyr-injected rats) and *n* = 20 (AAV-EV-injected rats). Because a randomly selected group of animals was euthanized for histological analyses at each time-point following the cylinder test, at the end of the behavioral analysis (24 m): *n* = 6 (AAV-hTyr-injected rats) and *n* = 4 (AAV-EEV-injected rats) animals remained. For the experiments with TFEB, vehicle (*n* = 7), AAV-TFEB (*n* = 5), AAV-hTyr (*n* = 3), and AAV-hTyr + TFEB (*n* = 4) injected rats were analyzed 1 week before surgery (to establish the basal conditions for each animal) and then every 4 weeks until rats were killed 12 m post-injection. For the performance of the cylinder test, rats were first allowed to habituate to the experimental room for at least 1 h before each test. Then, rats were put in a glass cylinder and the total number of left and right forepaw touches performed within 5 min was counted. Data are presented as the percentage of the contralateral paw usage with respect to either AAV-EV-injected or control (vehicle and AAV-TFEB-injected) rats, as appropriate. Behavioral equipment was cleaned with 70% ethanol after each test session to avoid olfactory cues. All behavioral tests were performed during the light cycle by an investigator blinded to the experimental groups.

### In vivo microdialysis

To assess local effects of d-amphetamine sulfate and veratridine on striatal DA release in microdialysis experiments, drugs were dissolved in artificial cerebrospinal fluid (aCSF: 125 mM NaCl, 2.5 mM KCl, 1.26 mM CaCl_2_, and 1.18 mM MgCl_2_) and administered by reverse dialysis at the stated concentrations (uncorrected for membrane recovery). Stock solutions of veratridine were made in dimethyl sulfoxide and were diluted to appropriate concentrations in aCSF to reach 1% dimethyl sulfoxide. Concentrated solutions (1 mM; pH adjusted to 6.5–7 with NaHCO3 when necessary) were stored at −80 °C and working solutions were prepared daily by dilution in aCSF.

Microdialysis procedures in vehicle- (*n* = 6) and AAV-hTyr-injected rats (*n* = 6 for the Amphetamine experiment and *n* = 8 for the Veratridine experiment) were conducted essentially as described previously^76^. Rats were anaesthetized with sodium pentobarbital (60 mg/kg, i.p.) and implanted with 3-mm concentric dialysis probes (Cuprophan membrane, 6000 Da molecular weight cut-off) in the ipsilateral striatum at the following coordinates (in mm): AP, + 0.5; L, −3.0 and DV, 6.6 (ref. ^75^). Microdialysis experiments were performed in freely moving rats 24–48 h after surgery, except in experiments involving the electrical stimulation of the medial forebrain bundle (MFB) (see below). Probes were perfused with aCSF at 1.5 μL/min. Following an initial 100-min stabilization period, 5 or 7 baseline samples were collected (20 min each) before local drug application by reverse dialysis and then successive dialysate samples were collected. In the experiments examining the effects on striatal DA release of electrical stimulation of the MFB, rats (*n* = 4 animals per group) were anaesthetized with chloral hydrate and 10 min fractions were collected (flow rate 3.0 μL/min). Bipolar stimulating electrodes consisted of two stainless steel enamel-coated wires (California Fine Wire, Grover Beach, CA, USA) with a diameter of 150 μm, a tip separation of ~200 μm and in vitro impedances of 10–30 KΏ. A stimulating electrode was stereotaxically implanted in the MFB (AP, −4.8; L, −1.0; and DV, −8.2 mm) and secured to the skull with glue and dental cement. Constant current electrical stimuli were generated with a Grass stimulation unit S-48 connected to a Grass SIU 5 stimulus isolation unit. Two stimulating conditions were used: S1 (2.0 Hz, 0.1 mA, and 0.2 ms) and S2 (10 Hz, 0.1 mA, and 1 ms)^77^. The concentration of DA in dialysate samples was determined by HPLC with electrochemical detection (Hewlett Packard 1049, Palo Alto, CA, USA). Striatal dialysates were collected into microvials containing 5 μL of 10 mM perchloric acid and were rapidly injected into an HPLC. DA was amperometrically detected at 5–7.5 min with a LOD of 3 fmol/sample using an oxidation potential of +0.75 V. Microdialysis results are expressed as femtomoles per fraction (uncorrected for recovery) and are shown in figures as percentages of basal values (individual means of 5–7 pre-drug fractions).

### Tissular levels of DA and metabolites

DA, DOPAC (3,4-dihydroxyphenylacetic acid), and HVA (homovanillic acid) contents were determined by HPLC with electrochemical detection (+0.7 V) as previously described^78^. AAV-hTyr-injected rats (*n* = 8) were killed at 4 m post-AAV injection and their brains quickly removed and placed over a cold plate. Contralateral non-injected and ipsilateral AAV-hTyr-injected striatum and substantia nigra from each animal were carefully dissected out using a Rat Brain Matrix (TedPella, Spain), frozen on dry ice, and kept at −80 °C until assayed. Tissues were homogenized in 300 or 500 μl (for substantia nigra or striatum, respectively) of buffer containing 0.4 M perchloric acid, 0.1% sodium metabisulphite, 0.01% EDTA, and 0.1% cysteine and centrifuged at 12,000 × *g* for 30 min. Aliquots of supernatants were then filtered through 0.45 μm filters (Millex, Spain) and analyzed by HPLC as described. The mobile phase consisted of 0.1 M KH_2_PO_4_, 1 mM octyl sodium sulfate, 0.1 mM EDTA (pH 2.65), and 18% methanol. DA and their metabolites were separated on a Mediterranea Sea column (C18, 3 μm, 10 cm × 6.4 mm) (Teknokroma, ref TR010042, Spain).

### UPLC-MS/MS analysis

AAV-hTyr-injected rats (*n* = 6) were killed at 1, 2, or 4 m after AAV-hTyr injections and the brains quickly removed and placed over a cold plate. Contralateral non-injected and ipsilateral AAV-hTyr-injected ventral midbrain from each animal were dissected out, frozen on dry ice, and stored at −80 °C until analyzed. On the day of analysis, samples were homogenized with 300 µl/hemisphere of 250 mM formic acid (FA) and protein concentrations determined by bicinchoninic acid (BCA) Assay Kit (Pierce). Dopamine-1,1,2,2-d_4_ hydrochloride (DA-d_4_) was added as internal standard (IS) to a final concentration of 500 nM and samples were then centrifuged at 20,000 × *g* for 10 min at 4 °C, and the supernatant was filtered using an Ostro™ protein precipitation and phospholipid removal plate (Waters, USA) and injected in the UPLC-MS/MS system twice to analyze MIX1 (dopamine, 3-methoxytyramine and oxidized dopamine) and MIX2 (3,4-dihydroxyphenylacetic acid). A Waters Acquity™ UPLC system was coupled with a Xevo TQ-S triple quadrupole mass spectrometer with electrospray ionization interface (Waters). Instrument control, data acquisition, and analysis were performed using MassLynx V4.1 (Waters). The chromatographic separation was performed on a Waters Acquity™ HSS T3 (1.8 μm; 2.1 × 100 mm) column coupled to a Acquity™ HSS T3 VanGuard (100 Å, 1.8 µm, 2.1 mm × 5 mm) pre-column and a Acquity™ UPLC in-line filter (Waters). Column temperature was set at 40 °C and samples were maintained at 6 °C in the thermostatic autosampler. The mobile phase consisted of solvent A (methanol 100%) and solvent B (25 mM FA in water) at a flow of 0.4 ml/min with the following gradient profile: 0.5% B maintained for 0.5 min, 8% B at 2.6 min, 55% B at 2.9 min, 60% B at 3.3 min, 80% B at 4.3 min, 90% B at 4.4 min and maintained for 0.5 min, and 0.5% B at 5 min followed by 1 min of equilibration. The mass spectrometer detector operated under the following parameters: source temperature 150 °C, desolvation temperature 450 °C, cone gas flow 50 L/hr, desolvation gas flow 1100 L/h and collision gas flow 0.15 ml/min. Argon was used as the collision gas. The capillary voltage was set at 0.5 kV for MIX1 and at 2 kV for MIX2 detection. The electrospray ionization source was operated in both positive and negative modes, depending on the analyte. Multiple Reaction Monitoring (MRM) acquisition settings for the targeted metabolites are summarized in Supplementary Table 3. Oxidized dopamine was detected using the following parameters: MRM transition (m/z) 149.61 > 121.91; cone voltage 25 V; collision energy 25 eV; capillary voltage kV 0.5 kV. Two independent weighted (1/x) linear regression curves excluding the origin were constructed for oxidized dopamine with 5 calibration points spiked to ventral midbrain samples of control rats. Linearity was considered acceptable if R2 ≥ 0.99 and more than 75% of the residuals had a variability smaller than 20%. With respect to sensitivity, the limit of detection (LOD) was defined as the concentration level with the signal-to-noise ratio at 3, and the limit of quantification (LOQ) was defined as the concentration level with the signal-to-noise ratio at 10 (see parameters in Supplementary Table 4). They were calculated as 3 or 10 times the ratio between the standard deviation of the Y-intercept value and the slope of each calibration curve, respectively. Samples with a concentration between LOD and LOQ or bigger than LOQ were considered acceptable; samples with a concentration lower than LOD were considered as 0 nM. Oxidized dopamine standard (0.5 mM) was freshly prepared as previously described^44,79^. In brief, 100 µl of 1 mM dopamine dissolved in water was mixed with 100 µl of 2 mM KIO4 dissolved in 100 µM aqueous ammonium acetate buffer (pH 5.8) at RT with vigorous shaking for 1 min. Successive dilutions were made in 25 mM FA.

### Human post-mortem brain tissue

Paraffin-embedded midbrain sections (5 µm) from idiopathic Parkinson’s disease patients (PD; *n* = 10), incidental Lewy body disease cases (ILBD; *n* = 3) and age-matched control individuals (*n* = 6) were provided by the Neurological Tissue BioBank at IDIBAPS-Hospital Clinic (Barcelona). Detailed information about these subjects is provided in Supplementary Table 1. Informed written consent was obtained from all human subjects. All procedures were conducted in accordance with guidelines established by the BPC (CPMP/ICH/135/95) and the Spanish regulation (223/2004) and approved by the Vall d’Hebron Research Institute (VHIR) Ethical Clinical Investigation Committee [PR(AG)370/2014]. For intracellular NM quantification in human brain samples, three different clinical diagnosis groups were evaluated: control subjects (1436 cells analyzed in a total of 6 cases), ILBD cases (640 cells analyzed in a total of 3 cases) and PD patients (644 cells analyzed in a total of 10 cases). Standard H&E staining was performed on 5-µm-thick paraffin-embedded SNpc sections for each subject. Identification of DA neurons was ascertained by the visualization of unstained NM pigment. For each section, all NM-positive neurons were examined at an objective magnification of ×20. Quantifications of intracellular NM optical density were performed as above by an investigator blinded to the experimental groups.

### Endogenous brain tyrosinase gene expression

Total RNA from human postmortem substantia nigra brain tissue (*n* = 8 control individuals; mean age at death 77 ± 3.7 years; detailed information for each case can be found in Supplementary Table 1) was extracted using miRNeasy Micro Kit (Qiagen). Total RNA from mouse (*n* = 5) and rat (*n* = 8) dissected ventral midbrain tissue was extracted using the mirVana PARIS RNA and Native Protein Purification Kit (Thermo Fisher Scientific). RNA concentration was determined using a NanoDrop ND-1000 Spectrophotometer and RNA integrity was assessed by running the samples on an Agilent RNA 6000 Nano chip on an Agilent 2100 BioAnalyzer (Agilent Technologies). For human samples, 10 ng of total RNA were retrotranscribed and amplified using the Ovation Pico WTA System V2 (Nugen Technologies, Inc.). For mouse and rat, 0.5 µg of total RNA were retrotranscribed using oligo(dT) 12–18 primers (Thermo Fisher Scientific) and SuperScript™ III Reverse Transcriptase kit (Thermo Fisher Scientific). Quantitative real-time PCR (qPCR) was performed with 45–50 ng of cDNA per well in technical duplicates mixed with Taqman Gene Expression Master Mix (Applied Biosystems) and Taqman gene expression assays (human tyrosinase (Tyr) Hs00165976_m1; rat Tyr Rn01511409_m1; mouse Tyr Mm00495818_m1; human tyrosine hydroxylase (TH) Hs00165941_m1; rat TH Rn00562500_m1; mouse TH Mm00447557_m1; Applied Biosystems) using standard procedures in a 7900HT Fast Real Time Instrument (Applied Biosystems). Thresholds cycles (Cts) for each target gene were normalized to an endogenous reference gene (Gapdh Mm99999915_g1 / Rn01775763_g1, Rpl19 Mm02601633_g1 / Rn00821265_g1 or Rplp0 Mm00725448_s1 / Rn03302271_gH for rodent samples, and HPRT1 Hs02800695_m1, GUSB Hs00939627_m1, or RPLP0 Hs04189669_g1 for human samples). Water was included in the reaction as a non-template (negative) control. The relative expression was calculated with the ΔCt-method. Serial dilutions of a DNA fragment corresponding to the full-length hTyr transcript, obtained by enzymatic digestion from a pCDNA4-hTyr plasmid (kindly provided by T. Hasegawa), were used as template for a qPCR reaction of 3 replicates for each dilution with the assay Hs00165976_m1 to specifically detect human Tyr expression and correlate the number of Tyr molecules and the Ct values obtained.

### TR5TY6 neuroblastoma cell line

A stable inducible SH-SY5Y cell line expressing human tyrosinase (TR5TY6) under the transcriptional control of the T-Rex TM Tet-On system (Invitrogen) was provided by Dr. T. Hasegawa (Department of Neurology, Tohoku University, Sendai, Japan). Cell line was confirmed negative for mycoplasma contamination by routine PCR analysis. Cells were maintained in low-glucose (1 g/l) Dulbecco’s modified Eagle’s (DMEM) (Gibco) medium with penicillin/streptomycin, and the appropriate selection of antibiotics (7 µg/mL blasticidin and 300 µg/mL Zeocin, both from Life Technologies). Medium was supplemented with tetracycline-free fetal bovine serum (FBS) (Clontech) to avoid unwanted expression of the transgene. TR5TY6 cells were seeded for aSyn-PLA, flow cytometry and immunoblot analysis at 2.5 × 10^5^ or 10^6^ cells/plate in 24-, 12-, or 6-well plates, respectively. For PLA, cells were grown onto 12-mm slides coated with 50 µg/ml poly-d lysine (Sigma-Aldrich). Unless stated otherwise, 24 h after seeding cells were differentiated with 10 µM retinoic acid (RA) (Sigma-Aldrich) for 3 days, followed by 80 nM 12-O-tetradecanoylphorbol-13-acetate (TPA) (Sigma-Aldrich) for 3 extra days prior to hTyr induction with 2 µg/ml doxycycline (Sigma-Aldrich) for up to 6 days. Intracellular NM in differentiated hTyr-induced TR5TY6 cells was quantified at the indicated times as follows: (i) optical densitometry in fixed cells, examined under transmitted light at an objective magnification of ×40; each slide was divided in a grid, images were taken from 25 fields (50% coverage) and analyzed as described above for rat sections; (ii) spectrophotometry, by diluting 2 µl of cell lysate in a total volume of 200 µl PBS and measuring the absorbance at 405 nm with a ELx800 Absorbance Microplate Reader (BioTek Instruments, USA).

### Immunoblot

Differentiated TR5TY6 cells induced for 0, 1, 3, and 6 days were homogenized in RIPA buffer supplemented with protease inhibitors (Roche) and cell extracts clarified by centrifugation at 10,000 × *g* for 10 min at 4 °C. Protein concentrations were quantified using the BCA method and subjected to SDS-PAGE. Proteins were resolved in 10 or 15% polyacrylamide gels and transferred onto 0.45 µm nitrocellulose membranes (Amersham). Blocking with 5% milk powder in PBS was followed by overnight incubation at 4 °C with the primary antibodies mouse anti-tyrosinase (1:2000, Thermo Fisher Scientific, Cat#MS-800-P1), rabbit anti-Lamp1 (1:250, GeneTex, Cat#GTX19294), rabbit anti-LC3 (1:1000, Novus Biologicals, Cat#NB100-2220), guinea pig anti-p62 (1:1000, Progen, Cat#GP62-C) or mouse anti-β-actin (1:5000, Sigma-Aldrich, Cat#A5441). Incubation with the secondary antibodies donkey anti-rabbit, sheep anti-mouse (both 1:5000, from Amersham), and goat anti-guinea pig (1:1000, Santa Cruz Biotechnology) was performed for 1 h at RT. Band densitometry, normalized to β-actin expression, were measured using ImageJ image analysis software.

### Optical microscopy for NM

Cells fixed with 4% paraformaldehyde for 30 min at 4 °C were blocked with 3% NGS (Atom) and 0.1% (vol/vol) Triton X-100 (Sigma-Aldrich) in PBS solution. Hoechst 33342 (1:10,000, Thermo Fisher Scientific) was used for nuclei counterstain for 1 h at RT in 2% (vol/vol) NGS. Cells were coverslipped using Dako Cytomation Fluorescent Mounting Medium (Dako) and images acquired using standard filter sets either with an Olympus FSX100 microscope with a DP72 incorporated camera and FSX-BSW visualization software (Olympus, Germany) or with an Olympus FluoView™ FV1000 confocal microscope and FV10-ASW 4.2 visualization software.

### aSyn-proximity ligation assay

TR5TY6 cells were induced for hTyr expression for 0, 3, and 6 days and fixed in 4% paraformaldehyde for 30 min at 4 °C. Custom PLA probes were prepared by conjugating 20 µl of the Ab-2 mouse monoclonal aSyn antibody (1 mg/ml) (Thermo Scientific, Cat#MS-1572) with one vial of Duolink In Situ Probemaker containing either PLUS (Sigma-Aldrich, Cat#DUO92009) or MINUS (Sigma-Aldrich, Cat#DUO92010) oligonucleotides. PLA assay was done using the Duolink in Situ Detection Reagents Red assay protocol (Sigma-Aldrich, Cat#DUO92008) following manufacturer’s instructions. Briefly, fixed cells were incubated with 3 drops of tempered blocking solution for 30 min at RT, shaking at low speed. Slides were then washed with PBS once and each slide incubated overnight at 4 °C with 20 µl of a mixture of both PLUS and MINUS antibody-conjugated oligonucleotide PLA probes in a humidity chamber. Ligation was done for 30 min at 37 °C, followed by amplification for 90 min at 37 °C, both in a humidity chamber as well. Nuclei counterstaining was done with Hoechst 33342 (1:10,000, Thermo Fisher) for 2 min. Slides were then washed three times with Washing Buffer B for 10 min each, mounted using fluorescent mounting medium (Dako) and visualized using an Olympus FSX100 microscope with a DP72 incorporated camera and FSXBSW visualization software (Olympus, Germany). Quantification of aSyn-PLA signal was performed by measuring intracellular pixel intensity values for individual cells (OFF, *n* = 153; 3d, *n* = 201; 6d, *n* = 135) in all acquired images using ImageJ.

### Mitochondrial respiration

Oxygen consumption rate (OCR) was measured in TR5TY6 cells using an XF24 Extracellular Flux Analyzer (Seahorse Biosciences, Agilent Technologies, USA). Briefly, 10^5^ cells/well were plated in XF24 microplates, differentiated, and induced for hTyr expression for 0, 3, and 6 days as described above. 24 h prior to the experiment, the instrument was calibrated following the manufacturer’s instructions. Cells were incubated for 1 h in 675 µl respiration medium (DMEM base, Sigma-Aldrich, supplemented with 5.5 mM glucose, 31.6 mM NaCl, Phenol Red, and 2 mM GlutaMax, Thermo Fisher Scientific) at 37 °C in a CO_2_-free incubator. Following overnight calibration of the sensor cartridge, plates were run on the Seahorse XF24 flux analyzer to calculate OCR. Four initial measurements, corresponding to basal oxygen consumption (routine state, without respiratory chain inhibitors), were performed per experiment, with each measurement cycle consisting of 4 min agitation, 2 min stand, and 1.5 min measurement. After each run, cells were lysed in RIPA buffer (150 mM NaCl, 10 mM Tris, pH 7.2, 0.1% SDS, 1% Triton X-100, 1% deoxycholate, 5 mM EDTA, 1 mM PMSF) and OCRs normalized to protein concentration, with measurements made using BCA. Four independent experiments were performed, each in triplicates.

### Intracellular protein degradation assay

Total protein degradation in differentiated TR5TY6 cells was measured by pulse-chase experiments as previously described, with some modification^80^. Briefly, confluent TR5TY6 cells were induced for hTyr expression for 0, 3, and 6 days and labeled with [^3^H]-valine (2 μCi/ml; Hartmann Analytic) at 37 °C for 24 h. Cells were then extensively washed with medium and returned in complete growth medium containing an excess of unlabeled valine for a chase period of 5 h. Cells were cultured with or without lysosomal inhibitors (100 µM leupeptin, 10 µM pepstatin A, and 10 µM E64d) to assess the contribution of the lysosomal system to protein degradation. Triplicated aliquots of the medium at the indicated time were collected and precipitated with 20% trichloroacetic acid. Acid-soluble fractions were collected after vacuum filtration using a Millipore manifolder and were quantified using a LS6500 liquid scintillation counter (Beckman Coulter). Cells were then lysed with 0.1% NaOH and 0.1% Triton X-100, and the total protein concentration measured for normalization purposes and to measure total surviving cells at the end of the induction times. Proteolysis was measured using a LS6500 liquid scintillation counter (Beckman Coulter) and expressed as the percentage of the initial acid insoluble radioactivity (total protein) transformed to acid-soluble radioactivity (amino acids and small peptides) at the end of the incubation time. Lysosomal proteolysis was calculated as the proteolysis sensitive to lysosomal inhibitors. Three independent pulse and chase experiments were performed.

### Ubiquitin-proteasome system activity

Differentiated TR5TY6 cells induced for hTyr expression for 0, 3, and 6 days were lysed in 10 mM tris pH7.8, 1 mM EDTA, 5 mM Mg2Cl, 0.1% Triton X-100. Chymotrypsin-like activity was determined using 10 µg of total cell extracts in a total volume of proteasome activity buffer (10 mM tris pH7.8, 1 mM EDTA, 0.5 mM DTT, 5 mM Mg2Cl, 2 mM ATP), incubating for 60 min at 37 °C with 0.5 mM of the fluorogenic substrate Z-Leu-Leu-Glu-AMC (Z-LLE-AMC) (Enzo, Cat# BML-ZW9345). As control, all reactions were also performed in parallel in the presence of 50 µM MG132 inhibitor (Enzo BML-PI102). Fluorescence intensity was quantified by using the Flx800 multi-detection microplate reader (BioTek) with excitation and emission wavelengths at 360 and 460 nm, respectively. UPS activity at different time points is presented as percentage of the control (OFF cells), and represent the mean of three independent experiments each performed in triplicates.

### TFEB-induced lysosomal exocytosis (dot blot)

TR5TY6 cells were transiently transfected using Lipofectamine ™3000 Transfection reagent (Thermo Fisher Scientific) according to the manufacturer’s instructions. Briefly, 5 × 10^4^ cells were seeded into 6-well plates the day before transfection with 2.5 µg of the construct pCIP-Flag-TfebAA (a gift from Reuben Shaw, Addgene plasmid Cat#79014)) and 5 µl of P3000 reagent. Transfected cells were harvested with medium, centrifuged at 800 × *g* at RT and 150 µl of culture medium were spotted onto a nitrocellulose membrane (GE Healthcare) using the Mini-Fold®-1 Dot-Blot system (Whatman, Inc.). The membrane was blocked in 5% non-fat milk powder in PBS for 1 h at RT and incubated overnight at 4 °C with mouse anti-Lamp2 (H4B4) antibody (1:1000, Santa Cruz Biotechnology, Inc. Cat#sc-18822). Incubation with anti-mouse secondary antibody coupled to horseradish peroxidase (1:1000 in 5% non-fat milk powder/PBS; GE Healthcare, #NXA931V and #NA934V) was performed for 1 h at RT, followed by repeated washings with PBS. Immunoreactive spots were visualized using SuperSignal Femto Chemiluminescent Substrate (Pierce) according to the manufacturer’s instructions and quantified on an ImageQuant RT ECL imaging system (GE Healthcare). Lysosomal exocytosis at different time points is presented as percentage of the non-TFEB-transfected cells, and represent the mean of three independent experiments run in duplicates.

### Cell metabolic activity and ROS production

Metabolic activity was measured in differentiated TR5TY6 induced for hTyr expression for 0, 3, and 6 days. Briefly, cells were centrifuged at 800 × *g* for 5 min at 4 °C, resuspended in PBS and incubated at 37 °C and 5% CO_2_ for 15 min with 10 µM C_12_-resazurin (Thermo Fisher Scientific). C_12_-Resazurin is readily reduced in metabolically active cells to red-fluorescent C_12_-resofurin (*λ*excitation = 563 nm; *λ*emission = 587 nm). To quantify the production of ROS, TR5TY6 cells were loaded with freshly prepared 25 µM CellROX Green Reagent (*λ*excitation = 485 nm; *λ*emission = 520 nm) (Thermo Fisher Scientific), a fluorescent dye that measures ROS production independently of its potential origin or location, for 30 min at 37 °C and 5% CO_2_. Cells were afterwards washed and harvested for flow cytometry analysis. Fluorescence data acquisition was performed by recording 10,000 events in an LSR Fortessa flux cytometer (BD Biosciences) and analyzed with FCS Express Version 4 software. Intact cells were gated in an FSC/SSC plot to exclude small debris. Cell metabolic activity and ROS production at different time points are presented as mean fluorescence intensity, and represent the mean of three independent experiments each performed in triplicates.

### Transmission electron microscopy

(i) Animals: rats were perfused transcardially with 4% PFA and 0.1% glutaraldehyde (Merck Millipore) in Dulbecco’s phosphate-buffered saline (DPBS, Gibco). Brains were removed and post-fixed for 4 h at 4 °C in the same fixative, washed in DPBS and sectioned into 100-μm-thickness slices using a vibratome (Leica, Germany). Free-floating sections were blocked in 0.1% Triton X100 and 10% NGS in PBS for 1 h at RT, incubated with primary antibody rabbit anti-TH (1:1000, Calbiochem) in PBS, a secondary goat anti-rabbit antibody conjugated to biotin (1:1000, Vector Laboratories), and visualized using Immunopure ABC Peroxidase staining kit (Thermo Fisher Scientific) and DAB Peroxidase Substrate kit (Vector laboratories). The sections were post-fixed in 3% glutaraldehyde and in 1% osmium tetroxide in 0.1 M cacodylate buffer (pH 7.4), dehydrated in ethanol, and embedded in Spurr’s epoxy resin (Sigma-Aldrich). Survey sections (2 μm) and ultra-thin sections (around 70 nm) were cut with a diamond knife (Diatome) using an ultramicrotome Ultracut E (Reichert-Jung). Ultrathin sections were collected onto formvar-coated copper/gold grids, counterstained with 2% uranyl acetate and led citrate, and examined in a JEOL 1010 (tungsten filament) transmission electron microscope. Images were acquired with an Orius CCD camera using Digital Micrograph software (GATAN). (ii) Post-mortem human brain: human control samples were initially fixed in formaldehyde and post-fixed in 3% glutaraldehyde, embedded in epoxy resin. Semi-thin and ultra-thin sections were processed as above. Sections were examined at a Zeiss 10 A transmission electron microscope. (iii) TR5TY6 cells: confluent 10-cm culture plates containing differentiated TR5TY6 cells induced for hTyr expression for 0, 1, 3, and 6 days were washed with a fixative solution containing 2.5% glutaraldehyde (Merck Millipore) in 0.1 M DPBS and then fixed for 1 h at RT in the same fixative. Cells were afterwards gently scraped and centrifuged at 300 × *g* to get a visible pellet that was kept in fixative at 4 °C until forward processing. Cell pellets were post-fixed in 1% osmium tetroxide in 0.1 M cacodylate buffer (pH 7.4), dehydrated in ethanol, and embedded in Spurr’s epoxy resin (Sigma-Aldrich). Semi-thin and ultra-thin sections, counterstain, and image acquisition were done as described above for rat sections.

### Statistics

All values are expressed as the mean ± standard error of the mean (SEM). Statistical comparisons were performed with SigmaStat software (v4, Systat Software Inc, USA) using the appropriate statistical tests, as indicated in each figure legend. No statistical methods were used to pre-determine sample size but our sample sizes are equivalent to those reported in previous similar publications^81^. Outlier values were identified by the Grubbs’ test (i.e. Extreme Studentized Deviate, ESD, method) using GraphPad Prism software (v6, GraphPad Software Inc, USA) and excluded from the analyses when applicable. Selection of the pertinent statistical test for each experiment was determined after formally testing for normality. Accordingly, differences among means were analyzed either by 1- or 2-way analysis of variance (ANOVA), Kruskal–Wallis ANOVA on ranks, two-tailed *t*-test or Mann–Whitney rank sum test, as appropriate. When ANOVA showed significant differences, pairwise comparisons between means were subjected to Student–Newman–Keuls, Dunn’s or Holm-Sidak post-hoc testing for multiple comparisons, as appropriate. In all analyses, the null hypothesis was rejected at the 0.05 level.

### Reporting summary

Further information on experimental design is available in the Nature Research Reporting Summary linked to this article.

## Supplementary information

Supplementary Information

Reporting Summary

## Data Availability

Data relevant to the study is available from the authors on reasonable request.
