# Supplementary material for: Brain tyrosinase overexpression implicates age-dependent neuromelanin production in Parkinson’s disease pathogenesis
Source: Nat Commun. 2019 Mar 7;10:973. doi: 10.1038/s41467-019-08858-y (PMC6405777; doi:10.1038/s41467-019-08858-y)
Supplement: Supplementary file 1 — Supplementary Information [file 41467_2019_8858_MOESM1_ESM.pdf]

Brain tyrosinase overexpression implicates age-dependent neuromelanin production in Parkinson's disease pathogenesis

Carballo-Carbajal, Laguna et al.

Supplementary Information

Supplementary Figures 1-8

Supplementary Tables 1-4

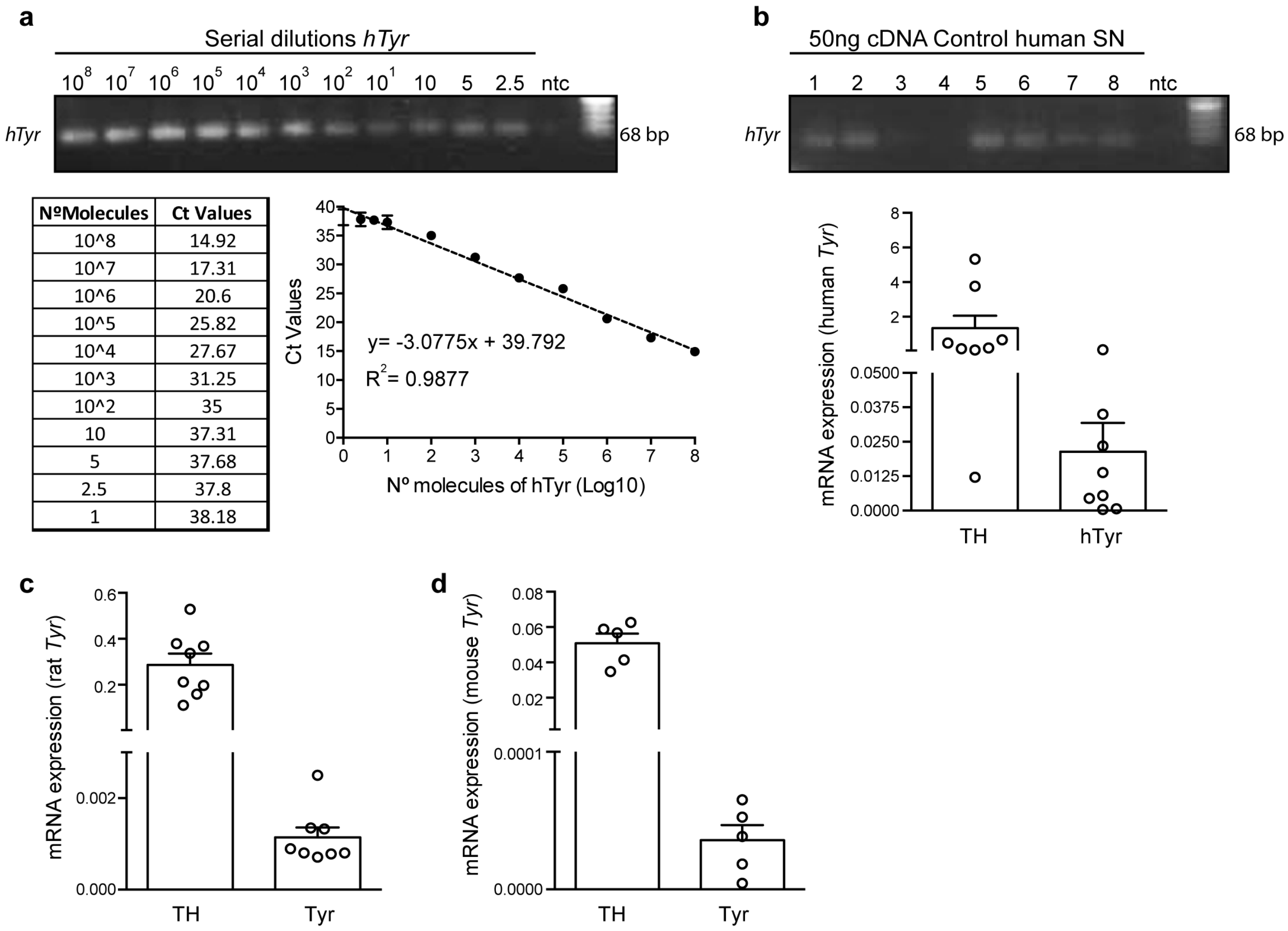

NºMolecules	Ct Values
10^8	14.92
10^7	17.31
10^6	20.6
10^5	25.82
10^4	27.67
10^3	31.25
10^2	35
10	37.31
5	37.68
2.5	37.8
1	38.18

Supplementary Figure 1. Tyrosinase expression in human, rat and mouse SNpc. (a-b) Detection of human *tyrosinase* (h*TYR*) gene expression by real-time reverse transcription-polymerase chain reaction (qPCR). **(a)** Serial dilutions of a DNA fragment corresponding to the full-length h*TYR* transcript, obtained by enzymatic digestion from a pCDNA4-hTYR plasmid (provided by Dr. T. Hasegawa (Department of Neurology, Tohoku University, Sendai, Japan)), were used as template for a qPCR reaction with the Taqman gene expression assay Hs00165976_m1 (Thermo Fisher Scientific) to specifically detect h*TYR* expression. The table represents the mean Ct Values obtained from the amplification of 3 replicates for each dilution (the number of molecules in each dilution is indicated) and the graph represents the correlation between the number of h*TYR* molecules and the Ct values obtained. **(b)** Measurement of h*TYR* gene expression using the comparative Ct method (2-delta Ct) in RNA samples from adult human postmortem substantia nigra (SN) brain tissue from control individuals (n=8; mean age at death, 79.3 ± 4.6 years; Supplementary Table 1). Values are normalized to an *HPRT1* endogenous reference gene. In two of the brains (#3 and #4), tyrosinase expression was below the detection levels. **(c-d)** Detection of rodent *tyrosinase (Tyr)* gene expression by qPCR in RNA samples from wild type (WT) adult rats (n=8) **(c)** and WT adult mice (n=5) **(d)** ventral midbrain tissues using Taqman gene expression assay Rn01511409_m1 and Mm00495818_m1 (Thermo Fisher Scientific), respectively. The comparative method (2-delta Ct) was applied to the mean of duplicate Ct values normalized to *GAPDH* as an endogenous reference gene. For comparison, normalized *tyrosine hydroxylase* (TH) gene expression values are also shown in **(b-d)**. Triplicate non-template controls (ntc; water) were performed as a negative control for all assays. Bars represent mean ± SEM. Individual values are shown as dot plots.

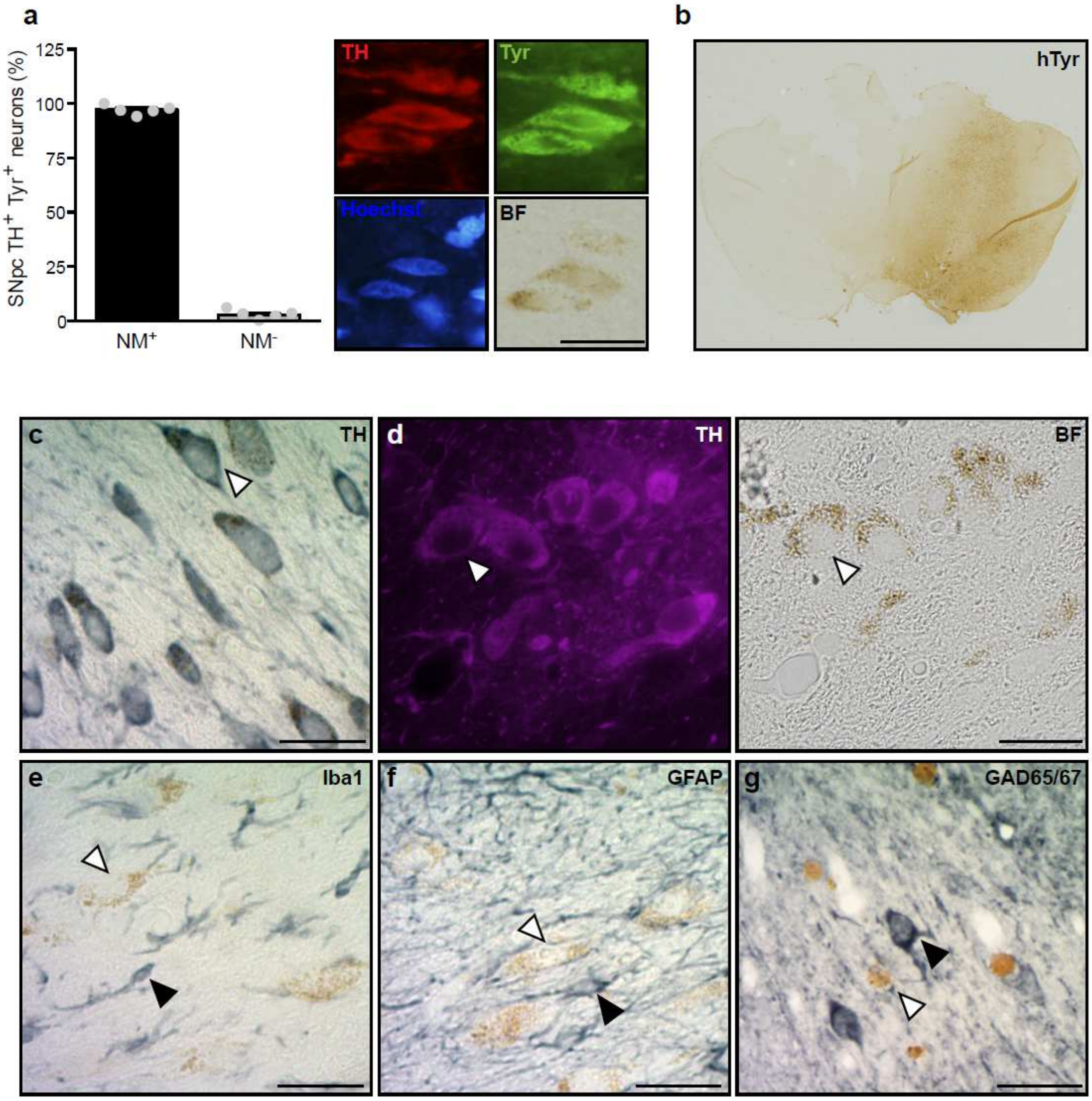

Supplementary Figure 2. NM production is restricted to TH-positive neurons in the SNpc of AAV-hTyr-injected rats. (a) Neuromelanin formation in hTyr-transduced neurons. Left, quantification of hTyr-expressing TH-positive neurons, with (NM+) or without (NM-) intracellular NM formation, from AAV-hTyr-injected rats at 1m post-AAV injection. An average of 82 neurons per animal at three different anatomical levels were analyzed (n=5 animals). Right, representative photomicrographs of a 30 µm-thick ipsilateral substantia nigra pars compacta (SNpc) section from an AAV-hTyr-injected rat (1m post-AAV injection) immunostained for TH (red) and hTyr (green). NM is seen in brown (BF, bright-field). Nuclei are visualized in blue. Scale bar, 15 µm. **(b)** Immunohistochemistry staining of hTyr (brown) from an AAV-hTyr-injected rat at 2m post-injection. **(c-g)** Despite the extensive expression of hTyr within the AAV injected hemisphere, NM production is restricted to TH-positive neurons in the SNpc of AAV-hTyr-injected rats as shown in representative photomicrographs of 5 µm-thick ipsilateral SNpc sections from AAV-hTyr-injected animals (2-4m post-AAV injection) immunostained for the DA neuron marker TH (**c**, blue; **d**, purple), microglial marker Iba1 (**c**, blue), astrocytic marker GFAP (**f**, blue) and GABAergic neuron marker glutamic acid decarboxylase GAD65+GAD67 (**g**, blue). In all panels, unstained NM is shown in brown. In **c**-**g**, white arrowhead point to a representative TH-positive neuron containing NM. In **e**-**g**, black arrowheads point to representative immunopositive cells (for the respective markers) lacking NM while white arrowheads point to representative NM-containing cells lacking immunostaining (for the respective markers). BF, bright-field. Scale bar, 25 µm.

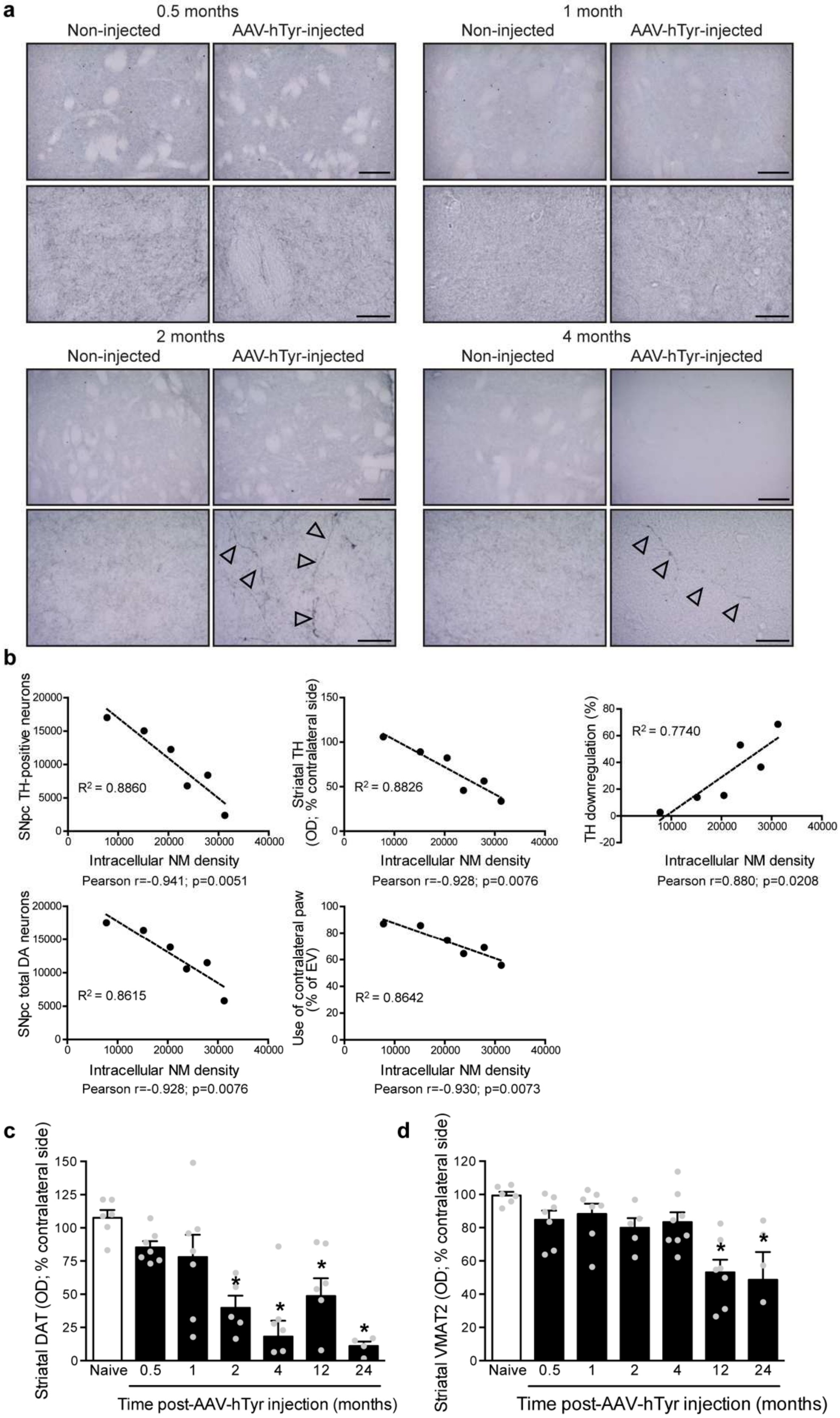

Supplementary Figure 3. Progressive PD-like degeneration of NM-laden SNpc DA neurons in AAV-hTyr-injected rats. (**a**) Representative photomicrographs of 30 µm-thick striatal sections from AAV-hTyr-injected rats immunostained for TH (blue) showing nigrostriatal axonal pathology in AAV-hTyr-injected rats. Arrowheads, abnormally enlarged nigrostriatal TH-positive nerve terminals. Scale bars: 150 µm (top panels); 25 µm (bottom panels). **(b)** Intracellular NM levels correlate with PD-related neurodegenerative changes in AAV-hTyr-injected rats. Pearson correlation analyses between SNpc intracellular NM optical density and PD-type neurodegenerative changes occurring in AAV-hTyr-injected rats (from left to right: number of SNpc TH-positive neurons, density of striatal TH fibers, TH downregulation within NM-laden neurons, total number of SNpc DA neurons and contralateral forepaw hypokinesia). Each point represents the average value for the corresponding parameter at any given time post-AAV-hTyr injection (0.5, 1, 2, 4, 12 and 24m). See main figures for *n* values per parameter and group. **(c-d)** Optical densitometry of striatal DAT **(c)** and VMAT2 **(d)** immunopositive fibers in AAV-hTyr-injected rats. *p<0.05, compared to naive animals (ANOVA on ranks; Dunn's post-hoc test). In all panels, values are mean ± SEM. n=6 (Naïve), n=7 (0.5m), n=7 (1m), n=5 (2m), n=8 (4m), n=7 (12m), n=5 (24m).

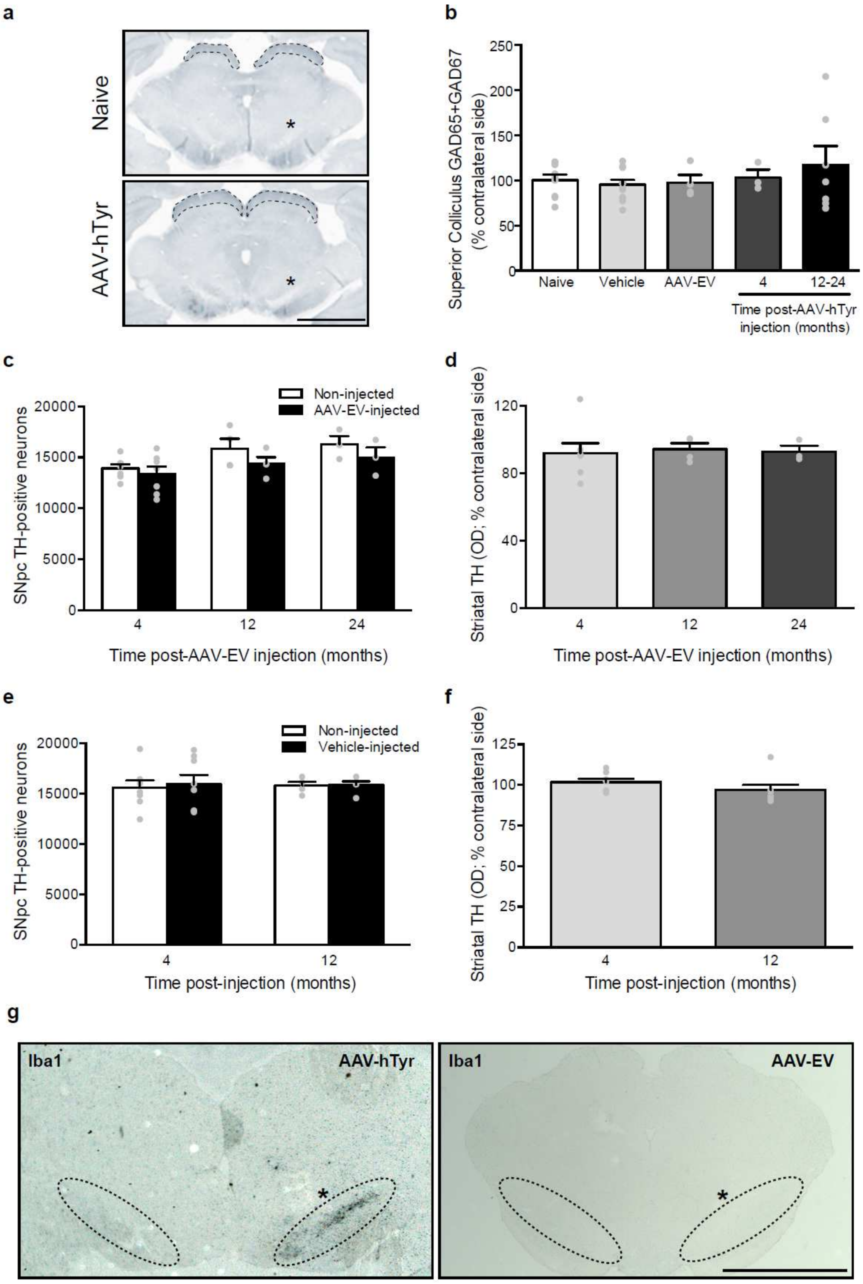

Supplementary Figure 4. Assessment of neurodegenerative and neuroinflammatory responses in AAV-hTyr-, AAV-EV- and Vehicle-injected rats. (a-f) Preservation of the GABAergic nigrotectal pathway in AAV-hTyr-injected rats and of the DA nigrostriatal pathway in AAV-EV- and vehicle-injected rats. **(a)** Representative photomicrographs of scanned 5 µm-thick midbrain sections immunostained for GAD65+GAD67 from a 12m-old naive rat and an AAV-hTyr-injected rat (12m post-AAV injection). Dashed outline, superior colliculus. Asterisks, injected side. Scale bar, 2mm. **(b)** Optical densitometry of superior colliculus GAD65+GAD67-positive fibers in naive (5-12m), vehicle-injected (4-12m post-injection), AAV-EV-injected (12-24m post-AAV-injection) and AAV-hTyr-injected (4-24m post-AAV injection) rats. **(c)** Stereological cell counts of SNpc TH-positive neurons in AAV-EV-injected rats. **(d)** Optical densitometry of striatal TH-positive fibers in AAV-EV-injected rats. **(e)** Stereological cell counts of SNpc TH-positive neurons in vehicle-injected rats. **(f)** Optical densitometry of striatal TH-positive fibers in vehicle-injected rats. In histograms, values are mean ± SEM. Individual values are shown in dot plots. In **b**, *n*=9 (naive), *n*=11 (vehicle-injected), *n*=4 (AAV-EV), *n*=3 (AAV-hTyr-injected at 4m) and *n*=7 (AAV-hTyr-injected at 12-24m); in **c**-**d**, *n*=7 (4m), *n*=4 (12m) and *n*=3 (24m); in **e**, *n*=8 (4m) and *n*=5 (12m); in **f**, *n*=8 animals per group. No statistically significant differences were detected between the different groups. **(g)** Lack of microglial-mediated neuronophagia in non-melanized SNpc from AAV-hTyr- and AAV-EV-injected rats. Representative photomicrographs of 5 µm-thick midbrain sections from an AAV-hTyr-injected (*left*) or AAV-EV-injected (*right*) rat (2-4m post-AAV injections) immunostained for the microglial marker Iba1 (blue). Dashed outline, SNpc; asterisks, AAV-injected side; unstained NM, brown. Scale bar, 1 mm.

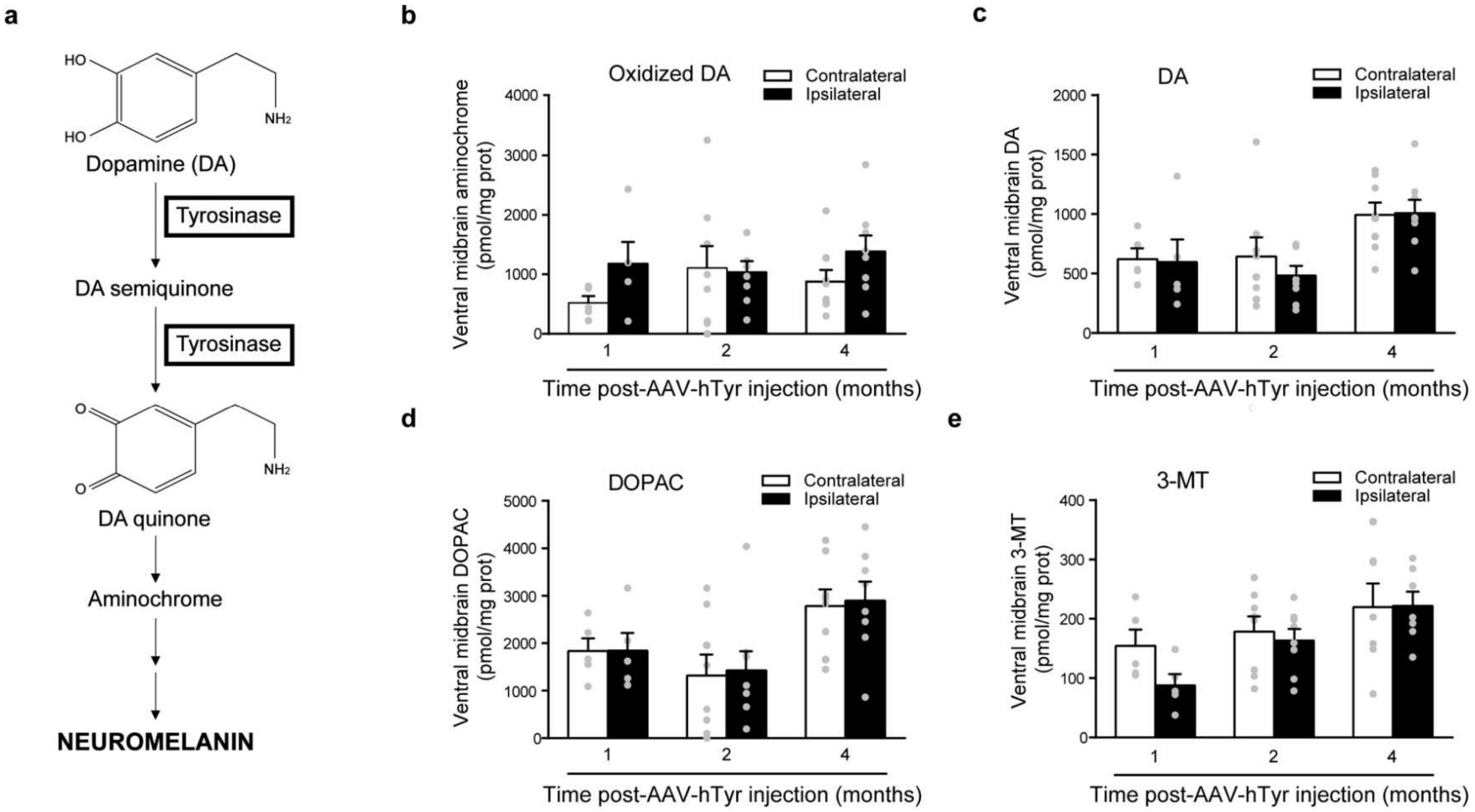

Supplementary Figure 5. Dopamine metabolite levels in the SNpc of AAV-hTyr-injected rats. **(a)** Schematic representation of tyrosinase-mediated production of dopamine oxidation metabolites. **(b-e)** Ventral midbrains levels (pmol/mg of tissue) of dopamine (DA) **(b)**, oxidized DA **(c)**, 3,4-dihydroxy-phenylacetic acid (DOPAC) **(d)** and 3-methoxytyramine (3-MT) **(e)** measured by UPLC-MS/MS in AAV-hTyr-injected rats at different times post-AAV injection. Data represent mean ± SEM. n=5 (1 month) and n=8 (2&4 months) animals per brain hemisphere. No statistically significant differences were detected between the contralateral (non-injected) and ipsilateral (AAV-hTyr-injected) ventral midbrain for the different metabolites analyzed (two-way ANOVA).

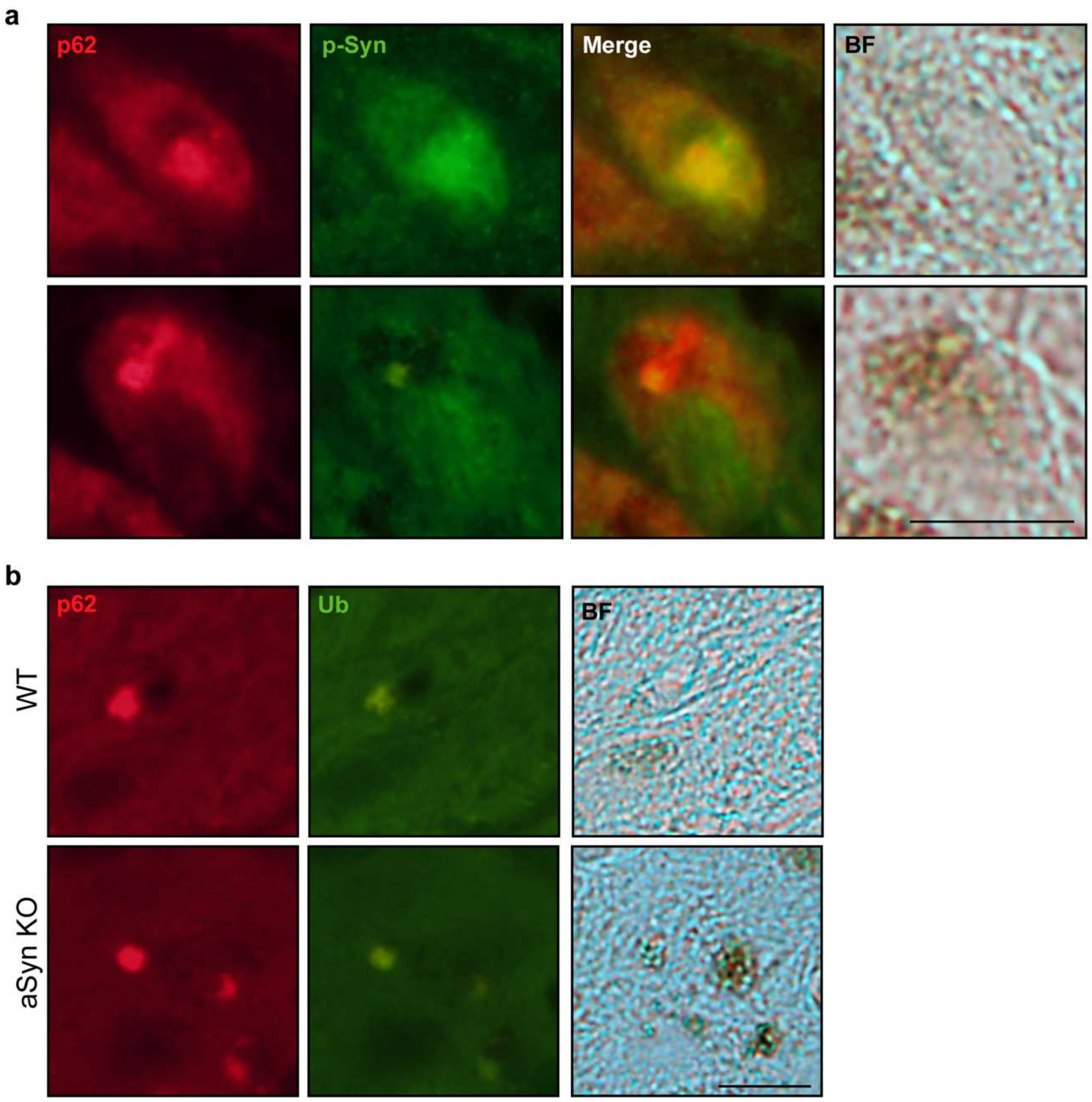

Supplementary Figure 6. PD-like inclusion body formation in NM-ladden neurons from NM-producing rats and ubiquitin-positive inclusions in aSyn-deficient NM-producing mice. (a) Representative photo-micrographs of 5 µm-thick ipsilateral SNpc sections from AAV-hTyr-injected rodents exhibiting intracytoplasmic PB-like (arrowhead, top) or LB-like (arrow, bottom) inclusions immunopositive for p62 (red) and S129 phosphorylated-alpha-synuclein (p-Syn, green). Unstained NM is shown in brown (bright-field, BF). Scale bar, 12.5 µm. **(b)** Representative photomicrographs of 5 µm-thick ipsilateral SNpc sections from AAV-hTyr-injected aSyn KO and WT mice exhibiting NM-laden neurons with cytoplasmic inclusions immunopositive for p62 (red) and ubiquitin (Ub, green). Unstained NM is shown in brown (bright-field, BF). Scale bar, 12.5 µm.

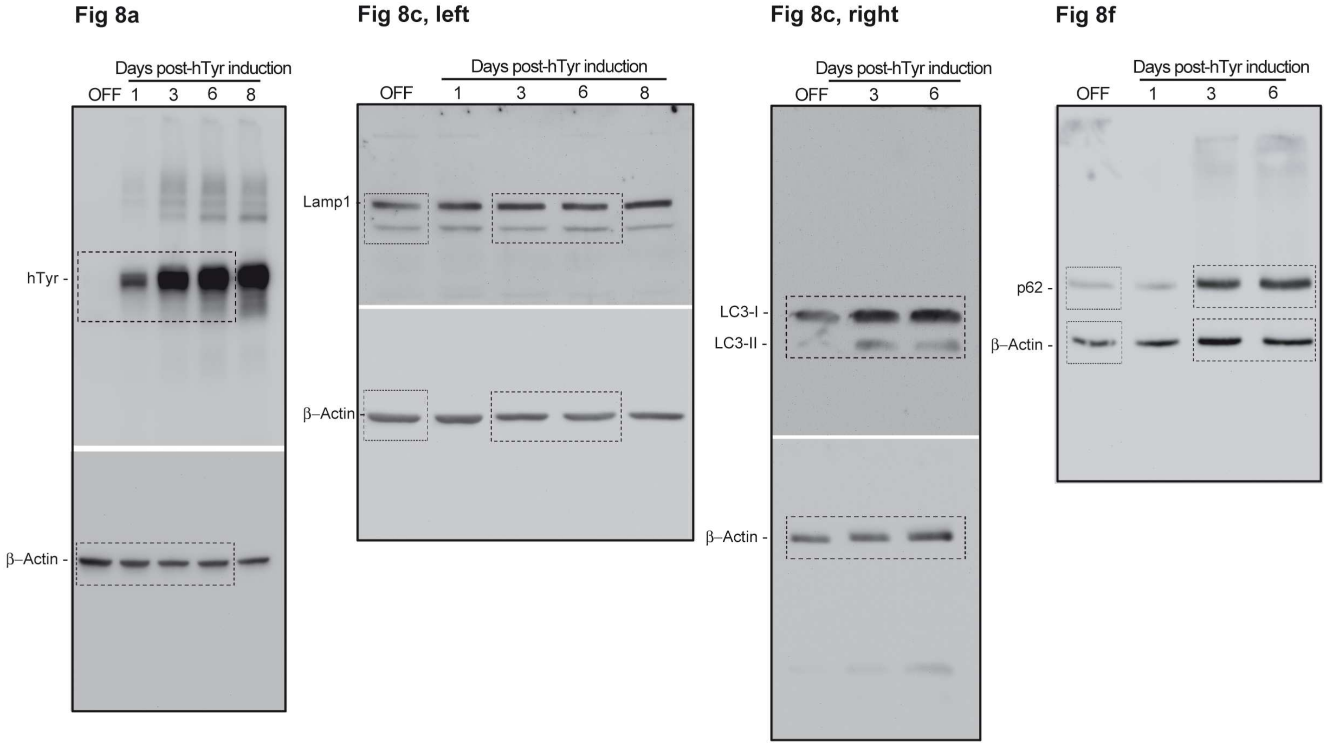

Supplementary Figure 7. Uncropped images of western blot figures shown in the main text (Fig 8). Dashed outlines indicate cropped sections.

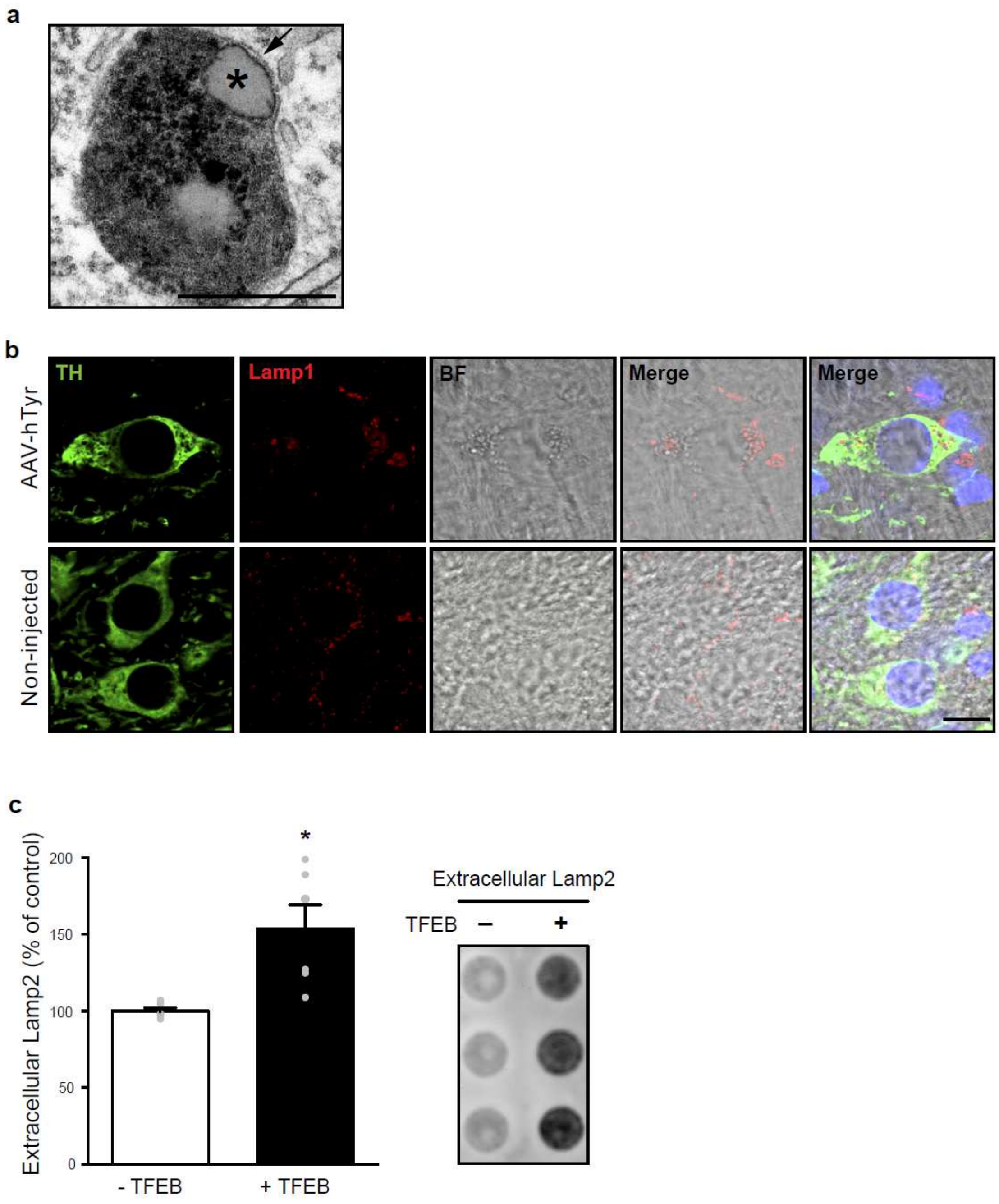

Supplementary Figure 8. Intracellular NM enclosed within autophagic structures in NM-producing rats and lysosomal exocytosis in TR5TY6 cells. (a) Representative electron micrograph of a NM granule in the ipsilateral SNpc of an AAV-hTyr-injected rat (4m post-AAV injection) showing a single-membrane-delimited vesicle (arrow) corresponding to mature lysosomes/autophagolysosomes containing electron dense intraluminal material (NM pigment) and characteristic lipid droplets (asterisk). Scale bar, 500 nm. **(b)** Top, confocal microscopy co-localization of the structural lysosomal marker Lamp1 (red) with NM granules (dark structures, brightfield) within ipsilateral dopaminergic (TH-positive, green) SNpc neurons of AAV-hTyr-injected rodents. Bottom, in the contralateral (non-injected) brain hemisphere, Lamp1 staining appears more scattered and clearly punctate within the cytoplasm of NM-free TH-positive neurons. **(c)** Dot-blot quantification of the lysosomal structural marker Lamp2 in the extracellular media of TFEB-transfected and non-transfected TR5TY6 neurons. Representative dot-blots are shown in the image. Values are mean ± SEM. N=6 per experimental condition. *p=0.002, compared to non-transfected cell media (Mann-Whitney rank sum test).

Supplementary Table 1. Human sample information

Case	Gender	Age at death (years)	Disease duration (years)	PMI (hr)	Clinical diagnosis	Braak PD-Staging (0-6) LB localization
Control-1	Female	91	-	8	No neurological disease	0-No LB
Control-2	Male	83	-	13	No neurological disease	0-No LB
Control-3	Female	86	-	4	Acute stroke	0-No LB
Control-4	Female	90	-	12.25	Acute stroke	0-No LB
Control-5	Male	58	-	5	Hepatic encephalopathy	0-No LB
Control-6	Male	76	-	11.5	No neurological disease	0-No LB
Control-7	Female	70	-	14	ELA stage I	0-No LB
Control-8	Male	70	-	4.5	Vascular Encephalopathy	0-No LB
Control-9	Female	83	-	7.2	No neurological disease	0-No LB
ILBD-1	Male	64	-	10	No neurological disease	1-Olfactory bulb
ILBD-2	Male	78	-	6	No neurological disease	1-Medulla Oblongata
ILBD-3	Male	86	-	10.25	No neurological disease	3-Brainstem

PD-1	Male	83	32	14	PD	5-Neocortex
PD-2	Female	88	12	16.5	PD	4-Basal prosencephalon-Mesocortex
PD-3	Female	85	16	7	PD	5-Neocortex
PD-4	Male	81	29	7	PD-D	5-6-Neocortex
PD-5	Male	77	1	7.5	PD-D	5-Neocortex
PD-6	Female	77	30	7.5	PD	5-Neocortex
PD-7	Female	81	8	3.75	PD-D	5-Neocortex
PD-8	Male	80	14	7.5	PD-D	6-Neocortex
PD-9	Male	77	21	6.25	PD	4-5-Mesocortex-Neocortex
PD-10	Male	76	22	12	PD	5- Neocortex

PMI: postmortem interval (hours); ILBD: incidental Lewy body disease; PD: idiopathic Parkinson's disease; PD-D: PD with dementia; LB: Lewy bodies.

Supplementary Table 2. Striatal and SN monoamine levels (pmol/mg of tissue) in AAV-hTyr-injected rats at 4m post-AAV injection

	**Dopamine**		**DOPAC**		**HVA**	
	Contralateral	Ipsilateral	Contralateral	Ipsilateral	Contralateral	Ipsilateral
Striatum	66.07 ± 10.13	38.40 ± 4.12*	10.44 ± 1.23	10.15 ± 1.04	4.28 ± 0.75	3.45 ± 0.29
SN	0.97 ± 0.06	1.08 ± 0.10	0.60 ± 0.06	0.44 ± 0.02*	0.33 ± 0.08	0.27 ± 0.02

DOPAC: 3,4-Dihydroxyphenylacetic acid; HVA: homovanillic acid; SN: substantia nigra.

Data represent mean ± SEM. *N*=8 animals per hemisphere and anatomical region. *$p<0.05$, compared to respective contralateral (non-injected) side (t-test).

Supplementary Table 3. Multiple Reaction Monitoring (MRM) acquisition settings for targeted metabolites and internal standard

Analyte	MRM transition (m/z)	MIX	Retention time (min)	Dwell (s)	Cone voltage (V)	Collision energy (eV)	Capillary Voltage (kV)
DA4d-95 (IS)	157,83 > 94,8	1-2	1,44	0,052	10	20	0,5
DA-91	153,93 > 90,57	1	1,46	0,052	10	20	0,5
3MT-91[a]	150,7 > 90,96	1	3,09	0,052	35	20	0,5
ODA-122	149,61 > 121,91	1	3,36	0,052	25	25	0,5
DOPAC-123[b]	166,99 > 122,82	2	3,72	0,056	18	22	2

DA4d: Dopamine-1,1,2,2-d4 hydrochloride; IS: internal standard; DA: dopamine; 3-MT: 3-methoxytyramine; ODA: oxidized dopamine; DOPAC: 3,4-Dihydroxyphenylacetic acid.

[a] Parent mass after loss of water

[b] Detected in negative mode

Supplementary Table 4. Oxidized dopamine sensitivity and linear range in ventral midbrain samples

**Brain region**	**Equation**	**R^2**	**Linear range (nM)**	**LOD (nM)**	**LOQ (nM)**
Ventral midbrain	Y = 0.01X + 0.56	0.9972	23.45 - 1500	4.41	14.72

LOD: limit of detection; LOQ: limit of quantification.
