# Supplementary material for: Brain tyrosinase overexpression implicates age-dependent neuromelanin production in Parkinson’s disease pathogenesis
Source: Nat Commun. 2019 Mar 7;10:973. doi: 10.1038/s41467-019-08858-y (PMC6405777; doi:10.1038/s41467-019-08858-y)
Supplement: Supplementary file 2 — Reporting Summary [file 41467_2019_8858_MOESM2_ESM.pdf]

nature research

Corresponding author(s): Miquel Vila Bover

Last updated by author(s): Dec 18, 2018

Reporting Summary

Nature Research wishes to improve the reproducibility of the work that we publish. This form provides structure for consistency and transparency in reporting. For further information on Nature Research policies, see Authors & Referees and the Editorial Policy Checklist.

Statistics

For all statistical analyses, confirm that the following items are present in the figure legend, table legend, main text, or Methods section.

n/a	Confirmed	
☐	☒	The exact sample size (*n*) for each experimental group/condition, given as a discrete number and unit of measurement
☐	☒	A statement on whether measurements were taken from distinct samples or whether the same sample was measured repeatedly
☐	☒	The statistical test(s) used AND whether they are one- or two-sided<br>*Only common tests should be described solely by name; describe more complex techniques in the Methods section.*
☒	☐	A description of all covariates tested
☐	☒	A description of any assumptions or corrections, such as tests of normality and adjustment for multiple comparisons
☐	☒	A full description of the statistical parameters including central tendency (e.g. means) or other basic estimates (e.g. regression coefficient) AND variation (e.g. standard deviation) or associated estimates of uncertainty (e.g. confidence intervals)
☐	☒	For null hypothesis testing, the test statistic (e.g. *F*, *t*, *r*) with confidence intervals, effect sizes, degrees of freedom and *P* value noted<br>*Give P values as exact values whenever suitable.*
☒	☐	For Bayesian analysis, information on the choice of priors and Markov chain Monte Carlo settings
☒	☐	For hierarchical and complex designs, identification of the appropriate level for tests and full reporting of outcomes
☐	☒	Estimates of effect sizes (e.g. Cohen's *d*, Pearson's *r*), indicating how they were calculated

Our web collection on statistics for biologists contains articles on many of the points above.

Software and code

Policy information about availability of computer code

Data collection	The data from this study was collected using the following software packages (as indicated in the corresponding methods sections): ZEN 2011, FV10-ASW 4.2, Digital Micrograph software (GATAN), Seahorse XF24 and Paravision 5.1.
Data analysis	The data from this study was analyzed with the following software packages (as indicated in the corresponding methods sections): SigmaStat v4, GraphPad v6, FCS Express v4, Image J, ZEN 2011, FSX-BSW, Cell Sens Entry v1.8, Digital Micrograph, Paravision v5.1, Stereoinvestigator v11 and SigmaScan Pro v5, FV10-ASW 4.2 and Seahorse XF24

For manuscripts utilizing custom algorithms or software that are central to the research but not yet described in published literature, software must be made available to editors/reviewers. We strongly encourage code deposition in a community repository (e.g. GitHub). See the Nature Research guidelines for submitting code & software for further information.

Data

Policy information about availability of data

All manuscripts must include a data availability statement. This statement should provide the following information, where applicable:

- Accession codes, unique identifiers, or web links for publicly available datasets
- A list of figures that have associated raw data
- A description of any restrictions on data availability

All data relevant to the study is available from the authors upon reasonable request.

Field-specific reporting

Please select the one below that is the best fit for your research. If you are not sure, read the appropriate sections before making your selection.

☒ Life sciences ☐ Behavioural & social sciences ☐ Ecological, evolutionary & environmental sciences

For a reference copy of the document with all sections, see nature.com/documents/nr-reporting-summary-flat.pdf

Life sciences study design

All studies must disclose on these points even when the disclosure is negative.

Sample size	Methods section / Statistical Analysis. For animal studies, sample size was chosen based on previous experience, on what would be manageable for the study, and on the anticipated variation according to previous experience from studies using related methods.
Data exclusions	In the cylinder behavioral test, rats that presented an asymmetric usage of the right-left paws at basal examination were excluded from the analysis (described in the Methods section).<br>Outlier samples were identified with GraphPad Prism v6 and excluded from the analysis (Methods section / Statistical Analysis).
Replication	All experimental findings were reliably reproduced. Each experiment was repeated independently 3 times and the values showed correspond to the mean value and SEM as indicated in Statistical Analysis Methods.
Randomization	Rats were randomly distributed into the different experimental groups and control and experimental groups were processed at once to minimize bias (Methods section / Animals).
Blinding	An observer blind to the experimental group performed the quantifications as indicated in the Methods section.

Reporting for specific materials, systems and methods

We require information from authors about some types of materials, experimental systems and methods used in many studies. Here, indicate whether each material, system or method listed is relevant to your study. If you are not sure if a list item applies to your research, read the appropriate section before selecting a response.

Materials & experimental systems

n/a	Involved in the study	
☐	☒	Antibodies
☐	☒	Eukaryotic cell lines
☒	☐	Palaeontology
☐	☒	Animals and other organisms
☐	☒	Human research participants
☒	☐	Clinical data

Methods

n/a	Involved in the study	
☒	☐	ChIP-seq
☐	☒	Flow cytometry
☐	☒	MRI-based neuroimaging

Antibodies

Antibodies used	Antibodies catalog numbers and additional information are given in the Methods sections for Immunohistochemistry, Immunoblot, and PLA assays.
Validation	All the antibodies used in this study have been previously reported and are commercially available. All of them have an online data sheet reporting the validity for the species and application tested.

Eukaryotic cell lines

Policy information about cell lines

Cell line source(s)	A stable inducible SH-SY5Y cell line expressing human tyrosinase (TR5TY6) under the transcriptional control of the T-Rex TM "Tet-On" system (Invitrogen) was provided by Dr. T. Hasegawa (Department of Neurology, Tohoku University, Sendai, Japan).
Authentication	The TR5TY6 neuroblastoma cell line has not been authenticated.
Mycoplasma contamination	Cell lines were tested negative for mycoplasm contamination. See methods section: Experimental models and subjects details - TR5TY6
Commonly misidentified lines (See ICLAC register)	No commonly misidentified cell lines were used.

Animals and other organisms

Policy information about studies involving animals; ARRIVE guidelines recommended for reporting animal research

Laboratory animals	Animal species, strain, sex and age are reported in the Methods section - Animals. In each figure legend, the age of the animals at sacrifice is specified.
Wild animals	The study did not involve wild animals.
Field-collected samples	The study did not involve samples collected from the field.
Ethics oversight	All animal experimental procedures were performed in strict accordance with the European (Directive 2010/63/UE) and Spanish laws and regulations (Real Decreto 53/2013; Generalitat de Catalunya Decret 214/97) on the protection of animals used for experimental and other scientific purposes, and approved by the Vall d'Hebron Research Institute (VHIR) Ethical Experimentation Committee.

Note that full information on the approval of the study protocol must also be provided in the manuscript.

Human research participants

Policy information about studies involving human research participants

Population characteristics	The population characteristics on human research participants is provided in Methods section: Human post-mortem brain tissue and Supplementary Table 1.
Recruitment	Participants were recruited at the Neurological Tissue Bank of the Biobanc-Hospital Clinic-IDIBAPS (Barcelona, Spain).
Ethics oversight	All procedures were conducted in accordance with guidelines established by the BPC (CPMP/ICH/135/95) and the Spanish regulation (223/2004) and approved by the Vall d'Hebron Research Institute (VHIR) Ethical Clinical Investigation Committee [PR(AG)370/2014].

Note that full information on the approval of the study protocol must also be provided in the manuscript.

Flow Cytometry

Plots

Confirm that:

- [ ] The axis labels state the marker and fluorochrome used (e.g. CD4-FITC).
- [ ] The axis scales are clearly visible. Include numbers along axes only for bottom left plot of group (a 'group' is an analysis of identical markers).
- [ ] All plots are contour plots with outliers or pseudocolor plots.
- [ ] A numerical value for number of cells or percentage (with statistics) is provided.

Methodology

Sample preparation	Cells were loaded with freshly prepared 25 µM CellROX Green Reagent (λexcitation=485 nm; λemission=520 nm) for 30 min at 37˚C and 5% CO2. Cells were afterwards washed with PBS and harvested for flow cytometry analysis. Methods section: Cell metabolic activity and production of reactive oxygen species (ROS).
Instrument	Data was acquired using a LSR Fortessa flux cytometer (BD Biosciences). Methods section: Cell metabolic activity and production of reactive oxygen species (ROS).
Software	Fluorescence data was analyzed with FCS Express Version 4 software. Methods section: Cell metabolic activity and production of reactive oxygen species (ROS).
Cell population abundance	Fluorescence data acquisition for positive or negative cells was performed by recording 10000 events from an homogeneous cell population. Methods section: Cell metabolic activity and production of reactive oxygen species (ROS).
Gating strategy	Intact cells were gated in an forward and side scatter (FSC/SSC) plot to exclude small debris. Gating of live cells was done using the viability dye C12-resazurin. Methods section: Cell metabolic activity and production of reactive oxygen species (ROS).

- [ ] Tick this box to confirm that a figure exemplifying the gating strategy is provided in the Supplementary Information.

Magnetic resonance imaging

Experimental design

Design type	2 months post-injection of AAV-Tyr in rat substantia nigra, brains were fixed and embedded in 2% agarose. "Ex vivo" 1H-

Design type	Magnetic resonance imaging (MRI) was done in whole brain, and the images used for qualitative assessment of neuromelanin presence in the injected side. Methods section: magnetic resonance imaging.
Design specifications	The acquisition time was 2h and 16 min for each high-resolution image. Methods section: magnetic resonance imaging.
Behavioral performance measures	N/A

Acquisition

Imaging type(s)	Structural
Field strength	Mini-imaging gradient set (400mT/m). Methods section: magnetic resonance imaging.
Sequence & imaging parameters	Parameter used for image acquisition are as follows (as indicated in the Methods section: magnetic resonance imaging: Low-resolution T2-weighted images: effective echo time (TEeff)=36 ms; repetition time (TR)=3 s; echo train length (ETL)=8; field of view (FOV)=6×6 cm2; matrix size (MTX)=128×128; slice thickness (ST)=2 mm; gap between slices (gap)= 0.5 mm; number of slices (NS)=25 -axial, 10 -sagittal, 11 -coronal; number of averages (NA)=1.<br>High-resolution T1-weighted spin-echo images: TE=9 ms; TR= 500 ms; NA=128; NS=20 (axial) and 7 (coronal); FOV=1.92×1.92 cm2; MTX=128×128 (axial) and 192×128 (coronal); ST= 0.25 mm (axial) and 0.35 (coronal);<br>Spatial resolution of 150×150×250 µm3 (axial) and 100×150×350 µm3 (coronal). The acquisition time was 2h and 16 min for each high-resolution image.
Area of acquisition	Area of acquisition was selected using initial low-resolution T2-weighted fast spin-echo images in axial, sagittal and coronal planes. High-resolution T1-weighted spin-echo images were acquired afterwards in the axial and coronal planes containing the region of interest. Methods section: magnetic resonance imaging.

Diffusion MRI ☐ Used ☒ Not used

Preprocessing

Preprocessing software	All MRI data were acquired and processed on a Linux computer using Paravision 5.1 software (Bruker BioSpin GmbH, Karlsruhe, Germany). Methods section: magnetic resonance imaging.
Normalization	Data were not normalized (images represent MRI qualitative evaluation)
Normalization template	N/A
Noise and artifact removal	Fixed rat brains were embedded in 2% agarose to diminish susceptibility artefacts. Methods section: magnetic resonance imaging.
Volume censoring	N/A

Statistical modeling & inference

Model type and settings	N/A
Effect(s) tested	N/A

Specify type of analysis: ☒ Whole brain ☐ ROI-based ☐ Both

Statistic type for inference (See Eklund et al. 2016)	N/A
Correction	N/A

Models & analysis

n/a	Involved in the study
☒	☐ Functional and/or effective connectivity
☒	☐ Graph analysis
☒	☐ Multivariate modeling or predictive analysis
